# A Systematic Study on Tooth Enamel Microstructures of *Lambdopsalis bulla* (Multituberculate, Mammalia) - Implications for Multituberculate Biology and Phylogeny

**DOI:** 10.1371/journal.pone.0128243

**Published:** 2015-05-28

**Authors:** Fangyuan Mao, Yuanqing Wang, Jin Meng

**Affiliations:** 1 Key Laboratory of Vertebrate Evolution and Human Origin of Chinese Academy of Sciences, Institute of Vertebrate Paleontology and Paleoanthropology, Chinese Academy of Sciences, Beijing, China; 2 Division of Paleontology, American Museum of Natural History, New York, New York, United States of America; Royal Belgian Institute of Natural Sciences, BELGIUM

## Abstract

Tooth enamel microstructure is a reliable and widely used indicator of dietary interpretations and data for phylogenetic reconstruction, if all levels of variability are investigated. It is usually difficult to have a thorough examination at all levels of enamel structures for any mammals, especially for the early mammals, which are commonly represented by sparse specimens. Because of the random preservation of specimens, enamel microstructures from different teeth in various species are often compared. There are few examples that convincingly show intraspecific variation of tooth enamel microstructure in full dentition of a species, including multituberculates. Here we present a systematic survey of tooth enamel microstructures of *Lambdopsalis bulla*, a taeniolabidoid multituberculate from the Late Paleocene Nomogen Formation, Inner Mongolia. We examined enamel structures at all hierarchical levels. The samples are treated differently in section orientations and acid preparation and examined using different imaging methods. The results show that, except for preparation artifacts, the crystallites, enamel types, Schmelzmuster and dentition types of *Lambdopsalis* are relatively consistent in all permanent teeth, but the prism type, including the prism shape, size and density, may vary in different portions of a single tooth or among different teeth of an individual animal. The most common Schmelzmuster of the permanent teeth in *Lambdopsalis* is a combination of radial enamel in the inner and middle layers, aprismatic enamel in the outer layer, and irregular decussations in tooth crown area with great curvature. The prism seam is another comparably stable characteristic that may be a useful feature for multituberculate taxonomy. The systematic documentation of enamel structures in *Lambdopsalis* may be generalized for the enamel microstructure study, and thus for taxonomy and phylogenetic reconstruction, of multituberculates and even informative for the enamel study of other early mammals.

## Introduction

Multituberculates are extinct mammals that lived in the Mesozoic and Paleogene and are the longest-lived group of mammals [1–3]. The oldest uncontested multituberculates are considered either from the Upper Jurassic [3] or from the Middle Jurassic [4–8]. The youngest members of the group are from Late Eocene [9–12]. Along with their long geological distribution, multituberculates are geographically widely distributed, known from all landmasses of the Northern Hemisphere [3]. If gondwanatherians were related to multituberculates, then they were also from landmasses of the Gondwana, including South America [13–17], Madagascar [16,18], and India [15]. Purported multituberculates were reported from the Cretaceous of Morocco [19,20], but they were regarded as haramiyidans by others [8,21]. More recent discoveries provided more robust evidence for presence of multituberculates in the southern continents [22–25], concurring their cosmopolitan distributions during the Mesozoic, although taxonomic positions of some of these forms still remain uncertain [3,25,26], partly because the specimens are fragmentary.

Multituberculates are generally considered to be a monophyletic group, characterized by having molars with multiple cusps arranged in at least two rows so that they are dentally distinctive from other mammals. Although they have long temporal and wide geographic distributions and are the best-known group among Mesozoic mammals [3], the majority of the multituberculate species was known from isolated teeth or fragmentary material. Thus, any morphological characters that potentially contain taxonomic and phylogenetic information are critical, among which the tooth enamel microstructures form a useful complex and can be obtained from limited material using non-destructive technique (un-embedded specimen) [22,27]. Moreover, tooth enamel microstructures have been used to infer evolutionary relationships and paleodiets in many other groups of mammals [27–33].

Tooth enamel microstructural study relies on the knowledge that enamel is composed of elongated, hexagonal crystallites of hydroxyapatite. Some crystallites are arranged in bundles as prisms and can be distinguished from others by different orientation of crystallites. The size, orientation, distribution and the packing patterns of crystallites and prisms are gene-controlled and have a limited range of intraspecific variability [31]. Enamel microstructural studies furnish useful, quantifiable, and reproducible data on dental tissues. A hierarchical system of classification for dental enamel of mammals, based on attributes of size, structural complexity, and distribution of enamel microstructures throughout the dentition, has been established [34,35].

In multituberculates, the gigantoprismatic and microprismatic enamels have been used to distinguish Ptilodontoidea and other post-plagiaulacoid multituberculates, [27,36]. These microstructures played a critical role in identifying taxa represented by limited specimens. For example, Kielan-Jaworowska et al. [22] observed that the holotype of *Argentodites coloniensis* from the Upper Cretaceous of Patagonia, Argentina, represented by a lower premolar (p4), having the normal, rather than gigantoprismatic enamel. This observation let the authors concluded that *A*. *coloniensis* has the affinity with Ptilodontoidea, although it differs from Laurasian cimolodontans in gross dental morphology.

As Carlson and Krause [27] demonstrated, the information for taxonomic interpretations of the microstructural data may be developed at a specified level of analysis. Ideally, the potential to facilitate accurate taxonomic and dietary interpretations using the enamel microstructures is best achieved if all hierarchic levels of enamel variability are investigated [27,31]. This is because variations of microstructures may exist in different locations of a tooth, in different teeth, or even in the way the specimens being prepared [34]. In reality, because of randomly preserved specimens, limitation of specimens and the destructive nature of the method, few studies have been done to examine all levels of enamel structures from a complete dentition of any mammals and the comparative studies of enamel microstructures have to be from different teeth and/or at a restricted level of enamel structures from various taxa [37–42].

Our question about the biological significance of the enamel is how much variation exists in the full dentition of a multituberculate species? The full dentition here means the deciduous incisors, the permanent upper and lower incisors, the upper and lower premolars and molars. Given the unique tooth morphology and function of the incisor, premolars and molars of multituberculates, the answer to this question is important to understanding the formation of the enamel as a biological process, the functional effect of the enamel as a mechanic feature in different teeth, and the level of reliability using the enamel microstructures to infer phylogenetic relationship. Extensive comparison has been conducted between species of many multituberculates [27] and some structures, such as prism density, has been sampled from several teeth of a single individual (from a mandible) for a few species, such as *Kryptobaatar daszevegi* [36], but a full survey on all levels of enamel structures from all teeth of one species has not yet been conducted.

Here we present a systematic survey of tooth enamel microstructures in *Lambdopsalis bulla*, a taeniolabidoid multituberculate from the Late Paleocene Nomogen Formation, Inner Mongolia. Taking the advantage of numerous specimens available in our collection, we have examined various regions of the full dentition. Using different methods, our observation ranges from crystallites, prism type (prism shape, prism size, prism density and enamel spindles/tubules, prism packing in the cross section), enamel types, Schmelzmuster and dentition types of the species. Our goal includes several aspects:

1. Provide a documentation of enamel structures and variability for the full dentition of a species (deciduous incisors, permanent upper and lower incisors, premolars and molars).
2. Explore various factors (sample locations at different tooth and treatment of the specimens, such as tooth sections cut in different orientations as well as acid etching at various degrees) that may affect the results of the enamel structures at all hierarchic levels as observed in various images (optic with ordinary and polarized light and SEM).
3. Discuss factors that may affect development of the tooth enamel structures as a biological process and the lifestyle or biology inferred from the enamel microstructures. We hope that the results may be generalized for other multituberculate species to understand the biological bases of the tooth formation and function, and contribute to the interpretation of the multituberculate evolution and phylogeny.
4. Take the advantage of digital publication to document the morphologies of enamel structures in detail and in color that were usually difficult in traditional publication.

## Materials and Methods

We sectioned a total of 32 teeth of *Lambdopsalis* that belong to 29 specimens, which are listed in Table 1. Two additional specimens that are used to show the boundary of root and enamel but not sectioned are not included in the table. These include: a right upper deciduous incisor (V 20715.1, Lower part of Nomogen Formation, Nuhetingboerhe, Erlian Basin, Inner Mongolia, China) and a left lower i2 (V 20716.1, Lower part of Nomogen Formation, Bayan Ulan, Erlian Basin, Inner Mongolia, China). These specimens and/or the resultant thin sections are housed in the Institute of Vertebrate Paleontology and Paleoanthropology (IVPP), Chinese Academy of Sciences, Beijing.

**Table 1:** Specimens of *Lambdopsalis bulla* sectioned in this study.

Specimen number	Tooth	Horizon and Locality
V 20297.1	Left di	Lower part of Nomogen Formation, Nuhetingboerhe, Erlian Basin, Inner Mongolia, China
V 20297.2	Right di	Lower part of Nomogen Formation, Nuhetingboerhe, Erlian Basin, Inner Mongolia, China
V 20297.3	Right i2	Lower part of Nomogen Formation, Nuhetingboerhe, Erlian Basin, Inner Mongolia, China
V 20297.4	Right m1-m2	Lower part of Nomogen Formation, Nuhetingboerhe, Erlian Basin, Inner Mongolia, China
V 20297.5	Broken right m1	Lower part of Nomogen Formation, Nuhetingboerhe, Erlian Basin, Inner Mongolia, China
V 20297.6	Left M2	Lower part of Nomogen Formation, Nuhetingboerhe, Erlian Basin, Inner Mongolia, China
V 20297.7	Right M2	Lower part of Nomogen Formation, Nuhetingboerhe, Erlian Basin, Inner Mongolia, China
V 20297.8	Right M2	Lower part of Nomogen Formation, Nuhetingboerhe, Erlian Basin, Inner Mongolia, China
V 20298.1	Left I2	Lower part of Nomogen Formation, Nuhetingboerhe, Erlian Basin, Inner Mongolia, China
V 20298.2	Right I2	Lower part of Nomogen Formation, Nuhetingboerhe, Erlian Basin, Inner Mongolia, China
V 20298.3	Right I2	Lower part of Nomogen Formation, Nuhetingboerhe, Erlian Basin, Inner Mongolia, China
V 20298.4	Right m1	Lower part of Nomogen Formation, Nuhetingboerhe, Erlian Basin, Inner Mongolia, China
V 20298.5	Left M1	Lower part of Nomogen Formation, Nuhetingboerhe, Erlian Basin, Inner Mongolia, China
V 20298.6	Right M1	Lower part of Nomogen Formation, Nuhetingboerhe, Erlian Basin, Inner Mongolia, China
V 20298.7	Left M2	Lower part of Nomogen Formation, Nuhetingboerhe, Erlian Basin, Inner Mongolia, China
V 20298.8	Right M2	Lower part of Nomogen Formation, Nuhetingboerhe, Erlian Basin, Inner Mongolia, China
V 20299–1	Right DI	Lower part of Nomogen Formation, Bayan Ulan, Erlian Basin, Inner Mongolia, China
V 20299–2	Right I2	Lower part of Nomogen Formation, Bayan Ulan, Erlian Basin, Inner Mongolia, China
V 20299–3	Right P4	Lower part of Nomogen Formation, Bayan Ulan, Erlian Basin, Inner Mongolia, China
V 20299–4	Right p4	Lower part of Nomogen Formation, Bayan Ulan, Erlian Basin, Inner Mongolia, China
V 20299–5	Right di	Lower part of Nomogen Formation, Bayan Ulan, Erlian Basin, Inner Mongolia, China
V 20300.1	Right di	Lower part of Nomogen Formation, Nuhetingboerhe, Erlian Basin, Inner Mongolia, China
V 20300.2	Right i2	Lower part of Nomogen Formation, Nuhetingboerhe, Erlian Basin, Inner Mongolia, China
V 20300.3	Right m2	Lower part of Nomogen Formation, Nuhetingboerhe, Erlian Basin, Inner Mongolia, China
V 20301.1	Left di	Lower part of Nomogen Formation, Nuhetingboerhe, Erlian Basin, Inner Mongolia, China
V 20301.2	Left i2	Lower part of Nomogen Formation, Nuhetingboerhe, Erlian Basin, Inner Mongolia, China
V 20301.3	Left p4-m2	Lower part of Nomogen Formation, Nuhetingboerhe, Erlian Basin, Inner Mongolia, China
V 20301.4	Right m1	Lower part of Nomogen Formation, Nuhetingboerhe, Erlian Basin, Inner Mongolia, China
V 20301.5	Left m2	Lower part of Nomogen Formation, Nuhetingboerhe, Erlian Basin, Inner Mongolia, China

Following the rule of specimen catalogue of IVPP, specimens from the same horizon and locality that belong to different individuals of the same species can be catalogued with the same number but followed with a different number after a decimal point “.”. Specimens that belong to the same individual must be catalogued with the same catalogue number and differentiated from each other with a number after a hyphen “-”.

No permits were required for the described study.

We observed the tooth enamel structures using an optic microscope with ordinary transmission light and polarized light and a scanning electron microscope. In preparing thin sections for optic microscopy, tooth samples were ultrasonically cleaned in water for 5–10 seconds and carefully placed on the pre-paved half solid DPX mounting medium so that the tooth orientation is certain after the mounting material is light cured. The mounted specimens were submerged in liquid DPX mounting medium and then vacuum pumped to remove air bubbles. The samples were exposed under a light-curing instrument (EXAKT 530) for 12 hours to have it set. The embedded tooth samples were mounted on glass slides and cut with a wire saw (EXAKT E300CP) with a thickness of 0.3mm. The sections were cut along the tangential, longitudinal and transverse directions to ensure different views of the enamel sample. The sections were grounded with 500 grit silicon carbide papers to smooth the cut surfaces and then polish with 1200 and 4000 grit silicon carbide papers to reduce scratches to a minimum. The final sections were approximately 100 μm thick, an optimum thickness with which the tooth sample displays clear details of microstructures under optic microscope. After being etched with 0.1 mol/L phosphoric acid in 50–70 seconds, the sections were examined using a binocular polarizing light microscope and digital images were captured with a Leica DMRX camera mounted on the microscope. The same sections, uncoated and coated (if needed), were also imaged for microstructures using a Hitachi S4700 Scanning Electron Microscope in the Key Laboratory of Vertebrate Evolution and Human Origins, Institute of Vertebrate Paleontology and Paleoanthropology, Chinese Academy of Sciences, Beijing, China.

### Observations

Patterns of enamel microstructure have been classified in a hierarchical system ranging from units describing the orientation and packing patterns of crystallites in small regions of a single tooth, to those identifying structural patterns of greater size and their distributions throughout the entire dentition of an individual organism. Five interdependent levels of enamel microstructures are distinguished [34], including: 1) Crystallites, orientation of crystallites; 2) Prisms type, cross-sections of prisms; 3) Enamel types, orientation of prisms relative to enamel dentine junction (EDJ) and differences in crystallite orientation of the interprismatic matrix (IPM) relative to prisms (these include radial enamel, tangential enamel, Hunter-Schreger Bands (HSB), and irregular decussation); 4) Schmelzmuster, three-dimensional arrangement of enamel types; 5) Dentitions, variation in Schmelzmuster throughout the dentition. The enamel structures at these levels in *Lambdopsalis bulla* are presented in separate sections below.

### Crystallites

**Fig 1:**
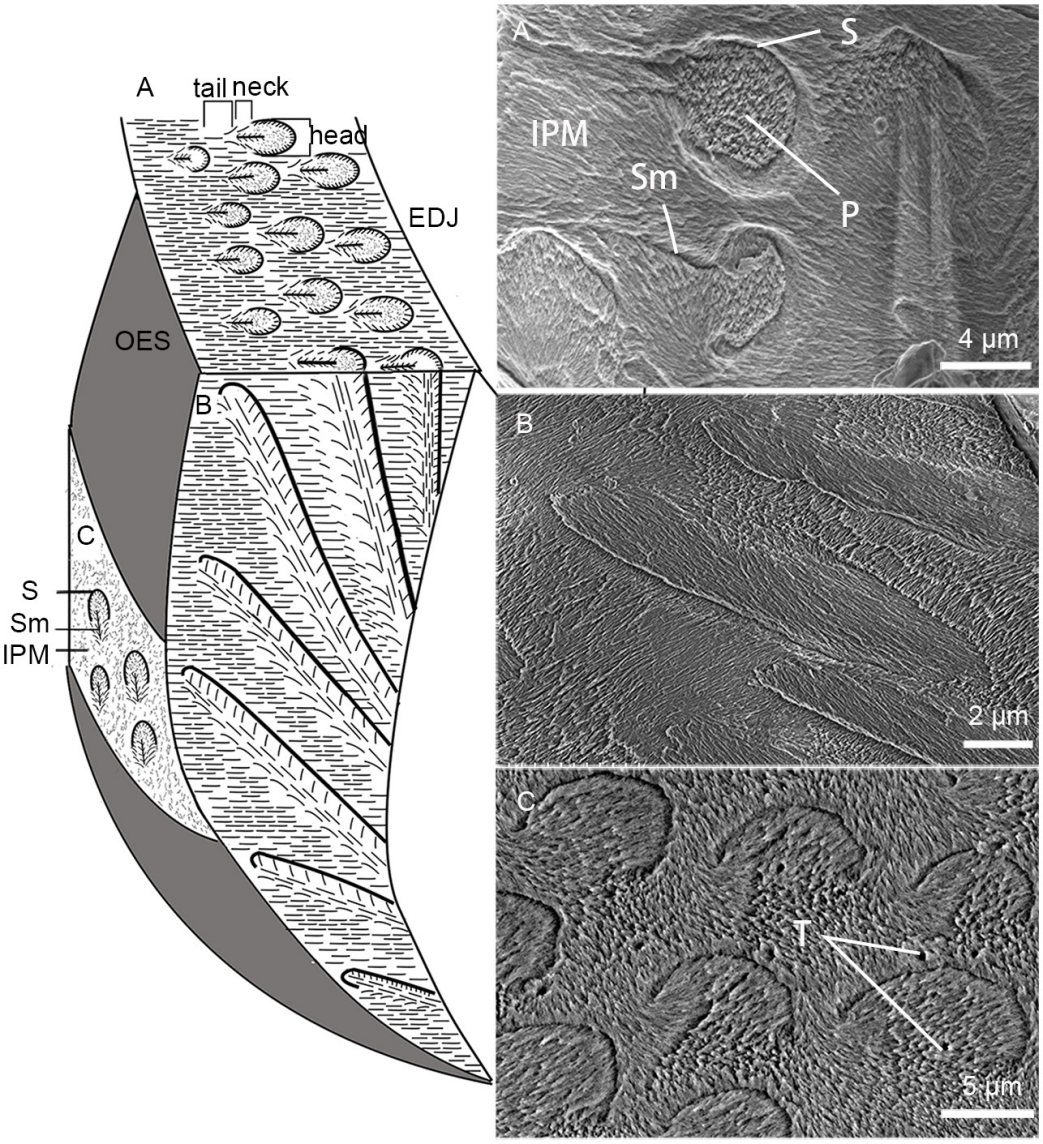
General pattern of crystallite orientation in tooth enamel of *Lambdopsalis bulla*. This pattern outlines the general enamel structures for all teeth with prisms that we examined, including incisors, premolars and molars. Sketch on the left shows the crystallite orientations on different sections of a sampled tooth enamel, including cross section (A), longitudinal section (B) and tangential section (C). The SEM-images on the right show the actual details of crystallite orientations on corresponding sections. The left sides of A and B are toward the OES and the right sides of them toward the EDJ. The upper side of C is towards the occlusal surface of a tooth and its bottom towards the cervical part of a tooth. Abbreviations: EDJ: enamel dentine junction; IPM: interprismatic matrix; OES: outer enamel surface; P: prism; S: sheath; Sm: seam; T: tubule.

The mass of mature enamel consists of 96% inorganic material and this component is comprised almost entirely of hydroxyapatite crystallites, which are the basic building blocks of enamel, either in prismatic or prismless enamel ([43]; Fig 1). Distinct from those of the prismless enamel types, the prismatic enamel is defined as having bundles of similarly orientated crystallites extended from the enamel dentine junction to almost the outer enamel surface without interruption and separation from other prisms by prism sheath or interprismatic crystallites [44]. The prism sheaths are planar crystallite discontinuity demarked the outlines of the prism, resulted from the sharp break on the shoulders of Tomes’ process in the secretory surface [45]. The crystallites of the prism sheaths show as differently oriented sets comparing to the crystallites of the prism. The crystallites of prismatic enamel type also form the IPM that separates the prisms (Fig 1).

The prism itself can be divided into three parts (best seen in cross section): the head, the neck and the tail ([46]; Fig 1). The distinction of the three regions of the prism is ambiguous in many cases and depends on species and orientation of the section where the prism is observed. Compared to early mammals, the prismatic enamel of most advanced mammals has denser prism sheaths but less extensive interprismatic areas [31]. In early mammals the prism usually has a linear or planar discontinuity termed prism seam in the middle of the prism tail and neck ([47–49]; Fig 1). Within the prismatic enamel, the size and orientation of the crystallites in the sheaths, IPM, prisms and around the seams, the crystallite orientation relative to each other and the variation in different parts of prisms (prism head, prism neck, prism tail, sheath, seam) play crucial parts in identification of microstructures of different enamel types [50,51].

For all permanent teeth with prismatic enamel in *Lambdopsalis*, crystallite orientations have approximately the same pattern, although the pattern may be altered by artificial treatment. In the cross sections of the cuspal enamel, where the cross section of prisms is best seen, the crystallites in the prism head are perpendicular to the planar of the prism cross-section and parallel with the long axis of the prism (Fig 1A). The crystallites bend toward the tail and show some angle with the long axis of the prism. The angle is gradually increased from the neck to the tail and reaches 60–70 degrees in the tail region. In the cross-section, the crystallites converge toward the seam and merge into the tail and the IPM.

**Fig 2:**
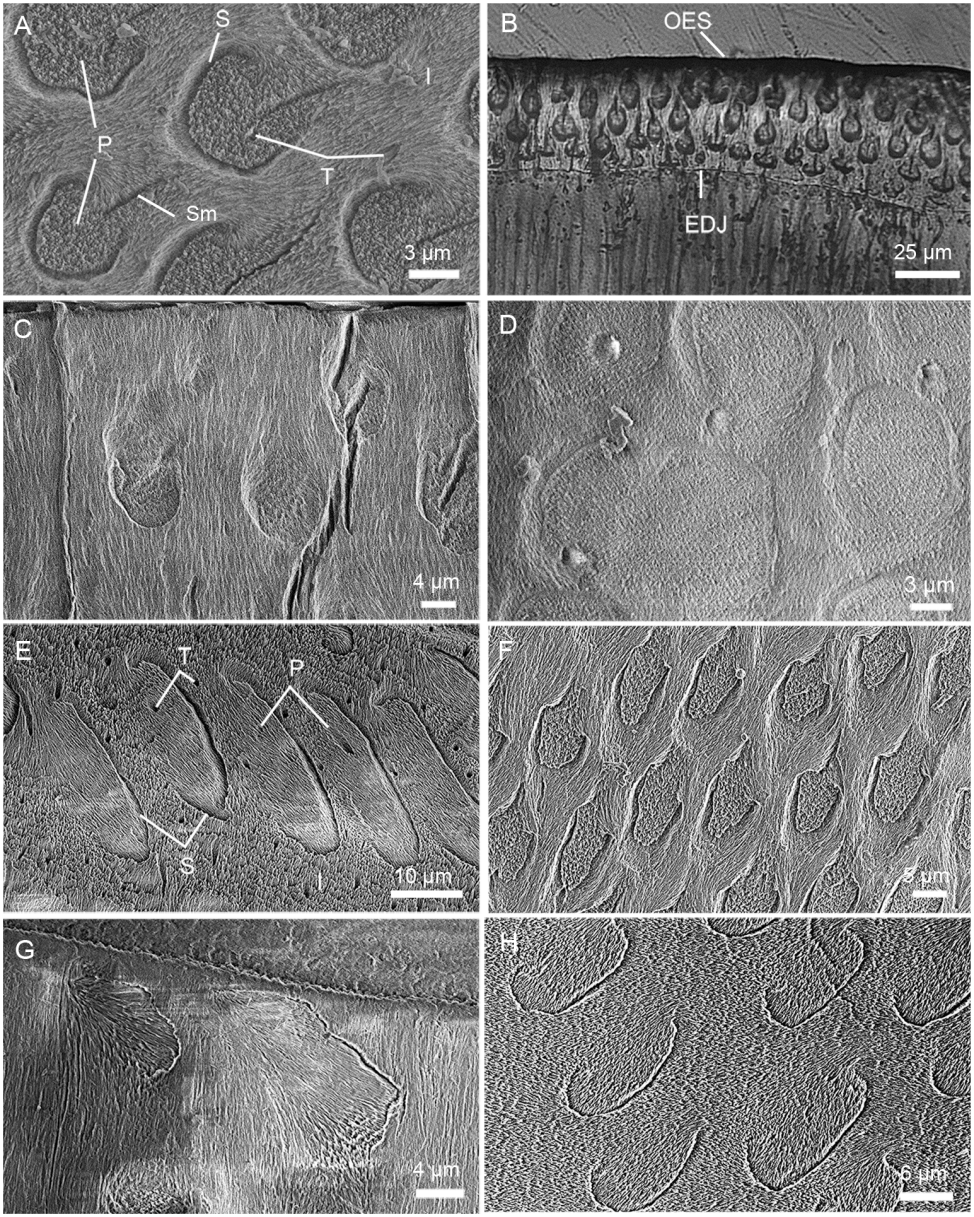
Enamel prism shape in different teeth of *Lambdopsalis bulla*. Cross-sections of (A) Left M2 (V 20298.7) and (B) Left M1 (V 20298.5) showing true outline of the sheath and shape of the seam; (C) The prism shape of the right di (V 20300.1); (D) Prisms of right m1 (V 20299–3) in insufficiently acid prepared section; E-H. Distortions of the prism shape caused by the cutting plane with an angle to the long axis of the prisms in the enamel of right I2 (V 20298.3), right m1 (V 20297.5), right p4 (V 20299–4) and right M2 (V 20298.8). All pictures are SEM-images except B, which is an optic image. Abbreviations: EDJ: enamel dentine junction; I: interprismatic matrix; OES: outer enamel surface; P: prism; S: sheath; Sm: seam; T: tubule.

It is not so easy to identify the orientation of crystallites in the sheath and/or prisms from the pretreated cross-section of prisms because the crystallites in these regions can be easily etched away after acid preparation. In the tooth enamel of extant vertebrates, acid preparation preferentially erodes the ends of crystallites faster than their longitudinal sides [52] and attacks the organic material stronger than the hydroxyapatite crystallites. However, in lightly etched tooth sections of *Lambdopsalis*, some sheath crystallites can still be preserved and visible (Fig 2D). The length of sheath crystallites is relatively shorter than those of the prism or IPM. They display as thin crystallite layers delimiting the outlines of prisms, where their orientations are nearly perpendicular to the surface of prism heads, the long prism axis and the crystallite direction of IPM, but parallel with the crystallites of the prism neck; these crystallites eventually fuse with the prism tail. As a result, the sheaths are clear near the heads of prisms but are not discernable at the necks and tails of prisms (Fig 1A).

The crystallites of the IPM tend to be orientated approximately parallel to each other and perpendicular to EDJ and the outer enamel surface (OES). The IPM crystallites form lamellae so that they appear to be linear in shape in the cross and longitudinal sections of the enamel (Fig 1A and 1B). In the tangential section, however, the crystallites usually appear as points or needle-like (Fig 1C). Within the IPM, there is variation in crystallite orientation at the junctions with tails of prisms. The crystallites in those areas are difficult to distinguish from those of the prism tails.

In cross-section of the prism in *Lambdopsalis*, the crystallites diverge toward the head but converge toward the tail, forming a somewhat symmetrical feather-shaped pattern with the seam being the stem. The seam bisects much of the prism within the sheath but rarely reaches the sheath. The seam extends through the open area of the sheath toward the OES for some distance and ends in the interprismatic crystallites (Fig 1). The length of the seams is unstable. In some prisms, the seams almost reach the adjacent prism sheaths, whereas other seams run only slightly out of the prism sheaths.

Comparative studies of enamel microstructures of amphibians, reptiles, and mammals indicate that, primitively, the crystallites of enamel do not form prisms, and tend to be approximately parallel in orientation; these crystallites extend radially outward from the EDJ toward the surface of the tooth [44]. This kind of the enamel is collectively termed as prismless enamel [53]. The approximately parallel-arranged crystallites are only found in some areas of various teeth, such as in the outer layer and cervical part of matured permanent teeth and in the enamel of the erupting deciduous incisor. This kind of enamel type was also called aprismatic enamel or parallel crystallite enamel [45,53], which we will further discuss in the following sections.

### Prism type

The prism type refers to a repeatable volume of enamel that is delimited by crystallite discontinuities or zones of crystallites with different orientation in the cross-section of prisms; it is used to describe characters of a prism that were considered as having some phylogenetic information [53].

Boyde [54] introduced a system to classify the morphology of prisms in cross-section and patterns of organization or packing of prisms. He recognized three patterns based on the size and shape of individual prisms and the two-dimensional array of a prism as revealed in the cross section of prisms. In pattern 1, the prism is completely surrounded by the prism sheath and commonly arranged in a hexagonal pattern. In pattern 2, the prism sheath is open basally, and prisms are arranged in approximately vertical rows separated by IPM. In pattern 3, prisms are also open basally but arranged in horizontal rows with alternating position. Pattern 2 and 3 are also termed arcade-shaped prisms.

With accumulating knowledge on the diversity of prismatic enamel Koenigswald and Clemens [34] considered that the system only focuses on the three major types of prism cross-sections and cannot cover the wide variety of the prism types. Moreover, correlations of these three patterns in different groups of vertebrates do not have too much congruity. Carlson and Krause [27] simply abandoned Boyde’s system in the study of multituberculate teeth because they found that the irregular spatial arrangement of prisms obscured the distinction between patterns 2 and 3, which made it difficult to decide the prism type in multituberculates. However, Gantt [55] expanded Boyde’s system into seven patterns in an effort to more precisely characterize prism types. The expanded system was widely used, although often informally, in the study of enamel microstructure of modern mammals [56–58].

At the prism level in this study we simply describe the morphologies of prisms and, wherever possible, use quantitative methods. We do not classify the prism type into the three patterns of Boyde [54]. Moreover, because the seam also exists in the enamel of some early mammal, multituberculates included, and is normally oriented perpendicular to the cross-section of the prism [48], therefore, the shape, length and orientation of the seam are also used to classify the prisms [52]. The structural complexity of enamel type may also increase by development of enamel tubule, which is considered as the termination of the dentinal tubules in enamel [34,46]. Thus, enamel tubule is treated as a feature in our description. To organize the description, we use five main categories of prism characters to describe and compare mammalian enamel prisms in the cross-section. These include the prism shape, prism size, prism density, enamel spindles/tubules, and prism packing in the cross section.

### 1) Prism shape

Our samples show that the enamel of all permanent tooth (incisor and cheek teeth) of *Lambdopsalis bulla* are prismatic, confirmed by both the scanning electron microscopy (SEM, Figs 1A and 2A) and the light microscopy (Fig 2B). The enamel of deciduous incisor also possesses the prismatic structure (Fig 2C). The prisms in these teeth have roughly the same structures and are interpreted as having both sheaths and IPM. In the enamel cross section of *Lambdopsalis*, the sheath is arch-shaped and accounts for nearly three-quarters of a circle with an open area facing the OES (Fig 2B). The prism sheath with the tail part open is consistent with the prism shape of the pattern 2 and 3 of Boyde’s system [54]. Contrasting with the circular prism (pattern 1 in Boyde’s system [54]), the orientation of crystallites on the open side of the prism gradually changes from the orientation of interprismatic crystallites (Figs 1A and 2A).

The seam is distinct in the enamel of all teeth in *Lambdopsalis*. It is quite slender compared to the round and broad head of the prism (Fig 2A). Usually the portion of a seam in the IPM is more slender than the rest in the prism head. The seam shape may have some variability in an individual tooth from the area near the EDJ to that of OES. The seam is shorter, slender and more uniform near the EDJ; it becomes thicker and longer toward OES; it gradually narrows down from the head to the tail and makes the head of the seam a tubule like opening.

The cross section parallel to the occlusal surface of the tooth best reveals the cross-sectional shape of the prism, but insufficient acid preparation may not reveal the true sheath and the seam (Fig 2D). This difference indicates that the seam and sheath contain crystallites or material that are less acid-resistant than the rest part of the prism. Except for various degrees of acid preparation, distortion of the prism shape may be caused by improper orientations of the specimen in preparation, as shown by prisms cross section with exaggerated width (Fig 2E) or length (Fig 2F). Moreover, the prism may display distorted shapes of the sheath and orientation of the prism and crystallites (Fig 2G and 2H). These are apparently resulted from cutting planes with various angles and orientations to the long axis of the prisms. Therefore, a meaningful comparison for prism shapes in different taxa strongly depends on how the specimens are prepared.

**Fig 3:**
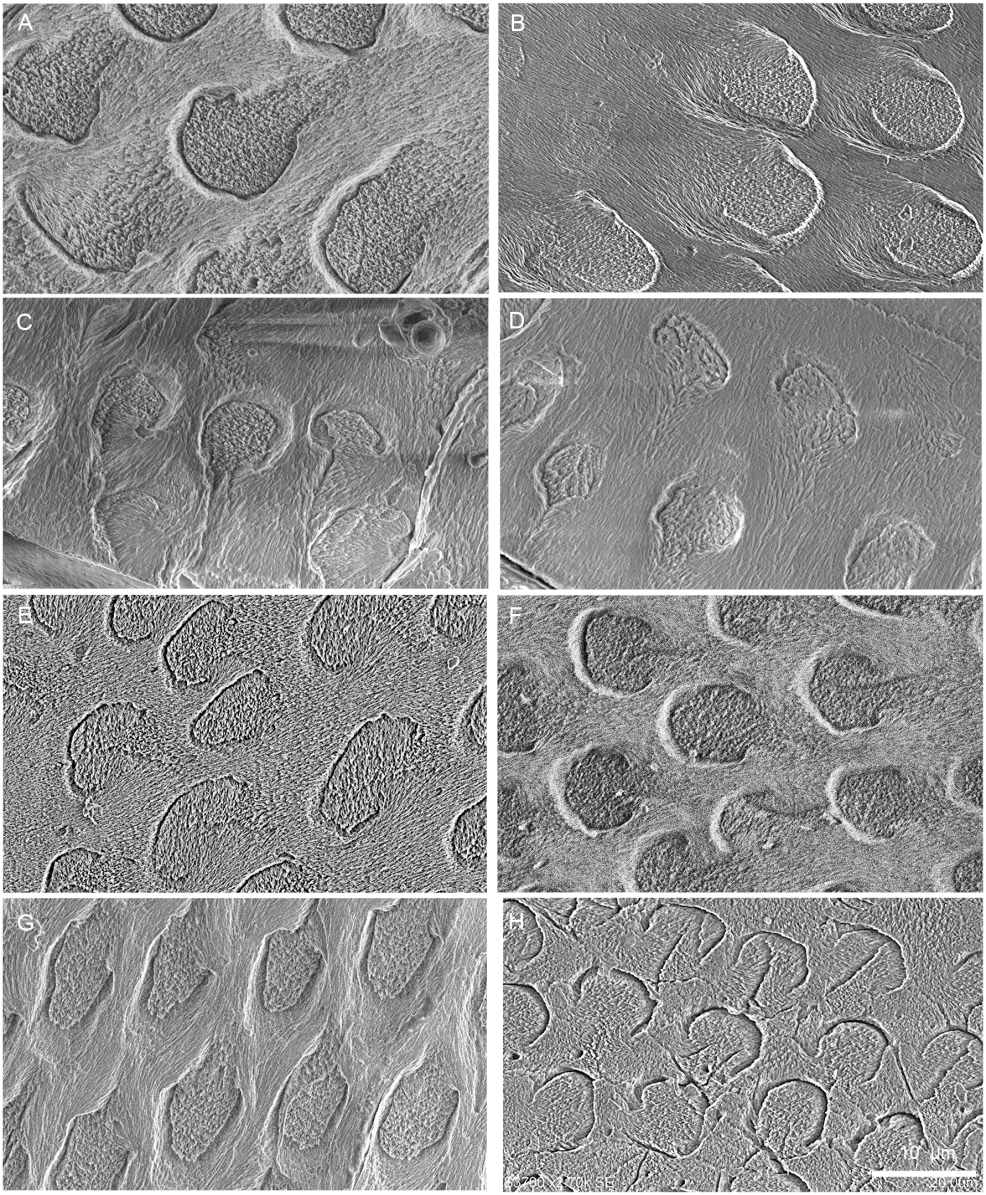
Prism shape in cross sections of different teeth in *Lambdopsalis bulla*. Although the prism shape in several pictures has some distortion, no distinct variation of the prism shape among prisms of different matured permanent teeth exist in the materials we examined. (A) Right I2 (V 20299-2); (B) Right i2 (V 20300.2); (C) Right P4 (V 20299-3); (D) Right p4 (V 20299-4); (E) Right M1 (V 20298.6); (F) Left M2 (V 20297.6); (G) Right m1 (V 20297.5); (H) Right m2 (V 20297.4).

With the potential instability in preparing the enamel microstructure in mind, we compared the prism shapes of all teeth in the tooth row of *Lambdopsalis bulla*. After numerous examinations of specimens cut at different orientations and acid prepared at different degrees, we are convinced that the cross section of the prism is arcade with the sheath forming nearly three quarters of a circle and the seam being distinct but quite slender. The result shows that the prism shapes among different matured permanent teeth are generally similar in *Lambdopsalis* (Fig 3). Similar prism shape is also present in deciduous incisors. However, because the prism size of deciduous teeth is relatively larger than that of permanent incisors, the prism shape appears more oval in the deciduous teeth.

**Fig 4:**
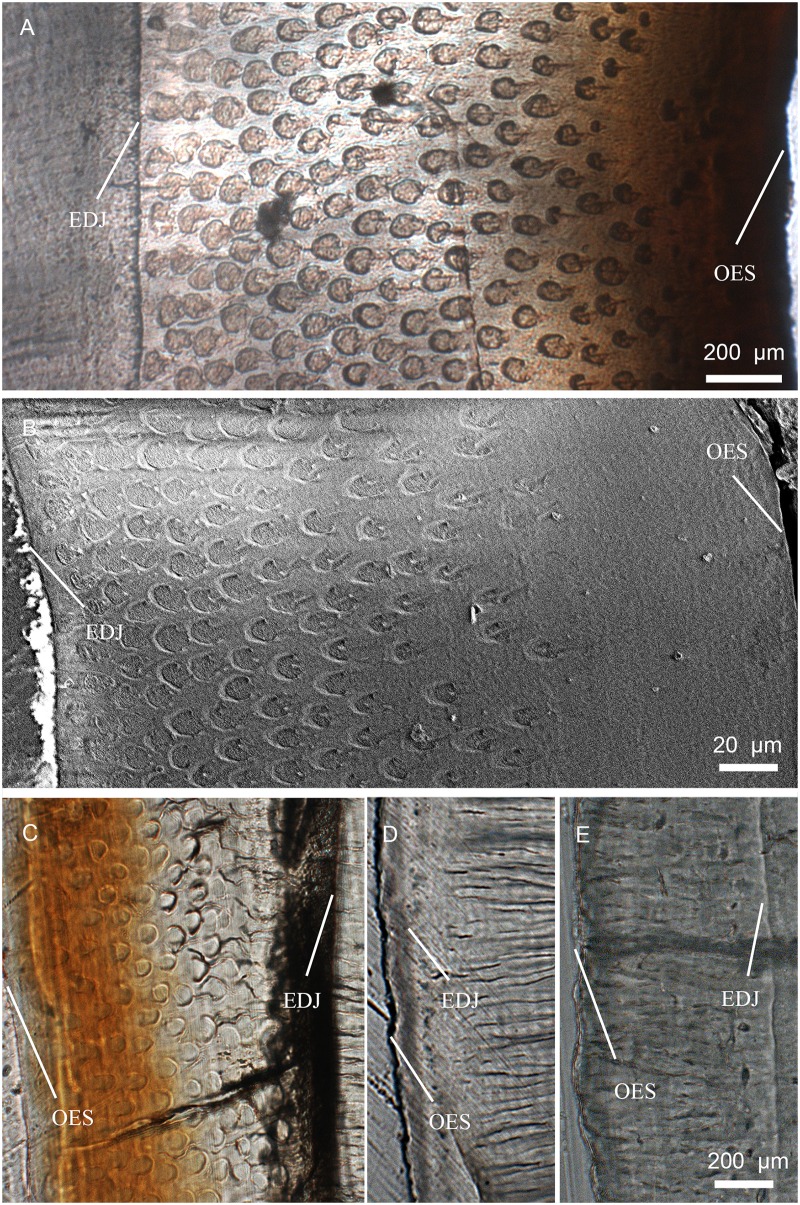
Variation of prism shape in tooth enamel of *Lambdopsalis bulla*. (A–B) Cross-sections of left M2 (V 20297.6) showing the atrophic prism in the outer part of mature enamel, Optic image (A) and SEM-image (B) for the same teeth; (C–D) Optic images of cross-section in different parts in the same right I2 (V 20298.2), cuspal area with prisms (C) and aprismatic enamel layer in cervical area of the tooth (D); (E) Optic image of cross-section in a right di (V 20300.1) with aprismatic enamel layer. C–E with the same magnification.

Although the arcade prism is common in most teeth of our samples, there is still variation of the prism shape in mature teeth of *Lambdopsalis*. It was known that a thin layer of aprismatic enamel is usually presented in the outmost part of the mature enamel of some early mammals [33]. Such an aprismatic enamel layer is clearly present in the outer layer of the mature tooth enamel of *Lambdopsalis*, but the boundary between it and the prismatic enamel is not clear-cut. The prism shape gradually changes from EDJ toward OES. In the same cross-section, the prisms in the inner layer are oval with the long axis parallel to the EDJ and have short seams, whereas those near the OES become more stretched in the direction perpendicular to the EDJ and have proportionally long seams relative to small sheaths (Figs 2B, 4A and 4B). Prisms in the middle portion of the enamel are more circular in outline. The size (diameter) of the prisms shows a gradual decrease toward the OES. In addition, the prisms near the EDJ and OES display some variations in structure, such as some sheaths are only in a half circle or in a complete circle and the prism seams are shorter or even absent.

**Fig 5:**
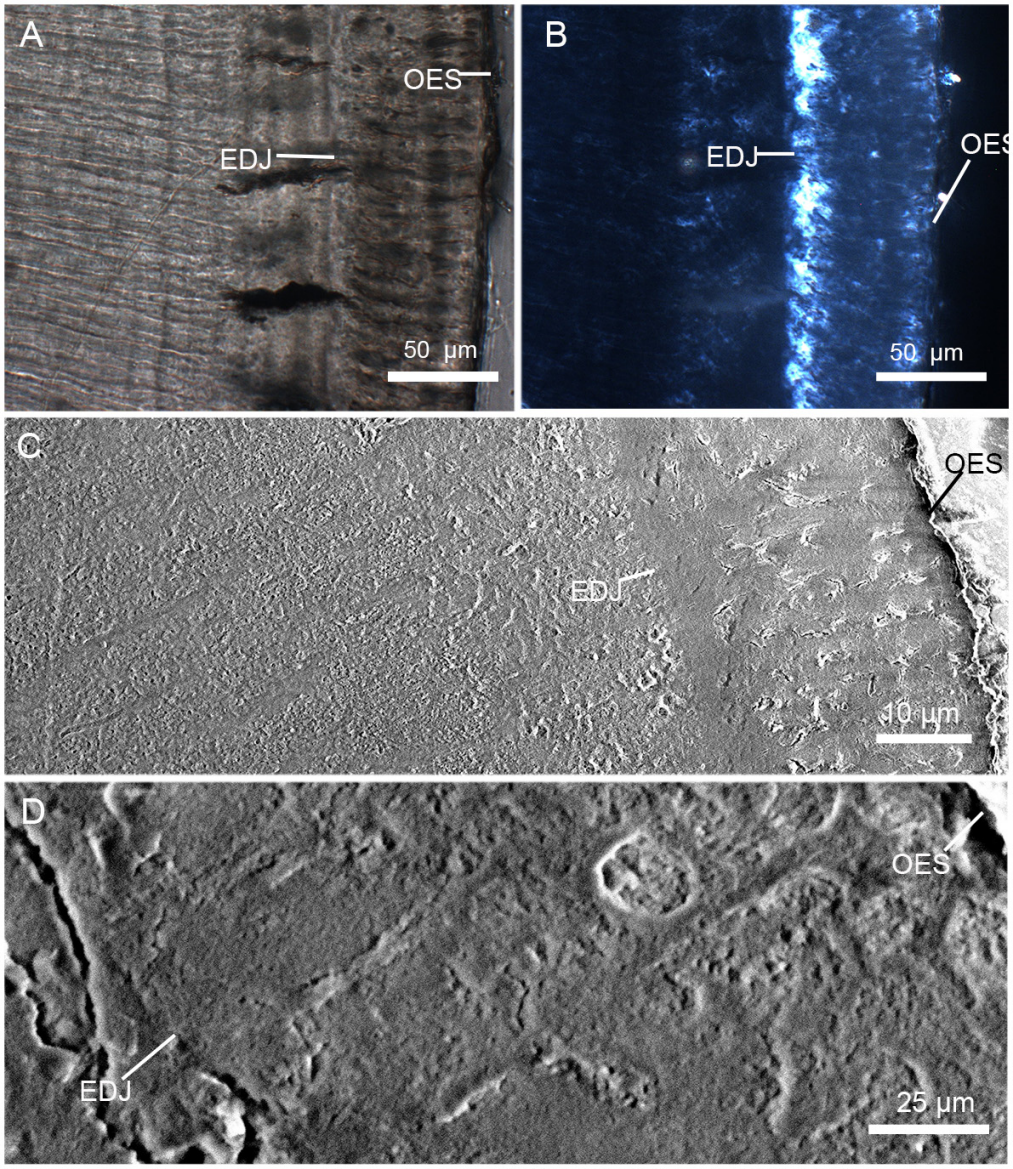
Cross section showing aprismatic layers in the cervical enamel of right DI (V 20299-1) of *Lambdopsalis bulla*. Aprismatic enamel is formed by apatite crystallites in columns that are clearer on the transmission light image (A); the EDJ is more distinct under polarized light (B); (C) Aprismatic enamel lacks the trace of the sheath and seam under SEM images; (D) Close-up view showing the enamel portion of (C).

Except for the prismatic enamel, the aprismatic enamel is frequently found in some portion of various teeth of *Lambdopsalis*; both may coexist in the same tooth (V 20298.2, Fig 4C and 4D). The aprismatic enamel is present in the cervical enamel of permanent teeth (V 20298.2, Fig 4D) and the enamel of the erupting lower deciduous incisor (V 20300.1, Fig 4E). Here we use aprismatic enamel to refer to the prismless enamel present in the outer layer of the tooth enamel in *Lambdopsalis*, knowing that the terminology has been used differently in various studies, as summarized in Koenigswald and Sander [53]. These aprismatic layers and areas account for a relatively small portion of the enamel in a permanent tooth. In the crown of permanent incisor (Fig 3A and 3B) and deciduous incisor (Fig 2C) the enamel always possesses arcade-shaped prisms with the seam. Differing from the prismatic enamel, the aprismatic enamel is formed by radial apatite crystallites (Fig 4D and 4E). Some of them seem orientated in columns (Fig 5A and 5B) but lacking the sheath between units and seam in the middle of units (Fig 5C and 5D), which was termed as the ‘synapsid columnar enamel’ (SCE) by Sander [45]. The EDJ in the aprismatic enamel is usually not very clear under ordinary transmission light (Figs 4D, 4E and 5A) but becomes distinct under polarized light (Fig 5B), probably owing to the difference in crystal structure and size between the enamel and the dentine.

### 2) Prism size

The cross section of prisms and the true prism diameter are clearer in the cross-section of teeth than in the longitudinal section of teeth. Any section tangential to the surface of the tooth reveals an accurate prism width, but the prism length (in cross section) may be artificially elongated. Circular prisms may appear oblong and arcade-shaped prisms may appear considerably longer than wide, depending on the orientation of the section. Thus, following Carlson and Krause [27], we measure the narrowest dimension of the prisms exposed in the section in order to minimize errors due to distortion of the prism outline caused by various orientations.

In our samples, the average prism diameter of *Lambdopsalis bulla* is 8.33 μm. However, variation of the prism diameter exists from the EDJ to the OES in the same section, as showed by Fig 4A and Table 2. We found that in general prisms near the OES have the shortest diameter, whereas those in the middle portion have the longest diameter (Fig 4A and 4B). The longest diameter can reach 13.7 μm, in contrast to the smallest one of only 4.83 μm (S1 Table). To reflect size variation from EDJ to OES, we divide the enamel cross-section into three zones with equal width as the inner, middle and outer zones, respectively. We then measured diameters of 20 prisms in a given area of each zone and the average diameter is used for the prisms in each zone. Due to the low density of prisms in some tooth (see below), in the corresponding area there may be fewer than 20 prisms, then the average diameter is calculated from the measured prisms. The actual measurements of the prism diameters are given in the S1 Table and the average diameters of prisms in three zones of different teeth are provided in Table 2.

**Table 2:** Prism size and prism density in different teeth of *Lambdopsalis bulla*.

Lower tooth	di			i2			p4			m1			m2		
Position	Inner	Middle	Outer	Inner	Middle	Outer	Inner	Middle	Outer	Inner	Middle	Outer	Inner	Middle	Outer
N	20	20	20	20	20	20	20	20	20	20	19	20	20	20	20
Average prism diameter	8.07	10.27	9.32	6.74	8.11	6.86	6.39	7.37	6.57	9.44	9.67	7.29	9.5	9.49	7.81
SD^a^	0.64	0.92	0.86	0.51	0.62	0.48	1.06	0.81	1.14	1.59	1.08	0.89	1.63	1.13	0.79
Prism density	2487	2246	882	7491	7782	3709	3078	2554	1637	4259	3127	1618	5186	4581	2968
d (SD^b^)	18.87 (3.91)			18.47 (3.67)			13.62 (2.55)			13.29 (2.52)			13.35 (2.19)		
Maximum prism density	3243			3385			6228			6536			6892		
Upper tooth	DI			I2			P4			M1			M2		
Position	Inner	Middle	Outer	Inner	Middle	Outer	Inner	Middle	Outer	Inner	Middle	Outer	Inner	Middle	Outer
N				20	20	20	15	20	20	20	19	20	20	20	20
Average prism diameter				9.71	10.45	7.02	6.45	7.17	6.63	7.6	10.56	9.33	10.69	10.84	6.7
SD^a^				1.16	1.54	1.08	0.57	0.6	0.73	0.9	0.88	0.99	1.19	0.96	1.15
Prism density				8268	7732	3445	4683	7713	6336	4468	5245	4338	6000	4267	1867
d (SD^b^)				17.47 (3.62)			12.54 (1.77)			10.64 (2.36)			12.94 (2.5)		
Maximum prism density				3785			7341			10193			6482		

Average prism diameter (in μm) is measured across the narrowest dimension exposed on the surface of the prism. N, number of prisms measured. Prism density is the prism number in per mm^2^. d, average mutual central distance between prisms. Maximum prism density (MPD) (the greatest number of prism per mm^2^) is calculated following the equation: MPD = (2×10^6^)/(d^2^)(3^(1/2)^) [53].

^a^, standard deviation about the prism diameter.

^b^, standard deviation about the mutual central distance between prisms.

The deciduous upper incisor (V 20299–1) we examined is an erupting tooth, which suggests that the specimen represents a very young individual. This is the only tooth in which we did not find any prism or similar structure in its enamel (Fig 5); thus prism size of this tooth is still unknown (Table 2).

The prism length is another dimension of its size, which is best revealed in the longitudinal section of the specimen. Although the length of prisms in different parts of a tooth may be approximately reflected by the thickness of the enamel, the accurate length is difficult to measure. This is because prisms are not absolutely straight and the entirety of prisms is seldom exposed (see Dentition types section), most of which are truncated by section plane. In general, the length of prisms is related to the thickness of the enamel so that in the cuspal area where the enamel is thick, the prisms are usually longer than that of the lateral enamel. For cheek teeth of *Lambdopsalis*, the prism length of M2/m2 is longer than that of M1/m1, whereas that of P4/p4 is the shortest. The prism length of the cheek teeth is longer than that of the incisors; the prism length of lower incisor is longer than that of the upper one. Finally, the prism length of the permanent incisor is longer than that of the deciduous incisor that has relatively thin enamel.

### 3) Prism density

**Fig 6:**
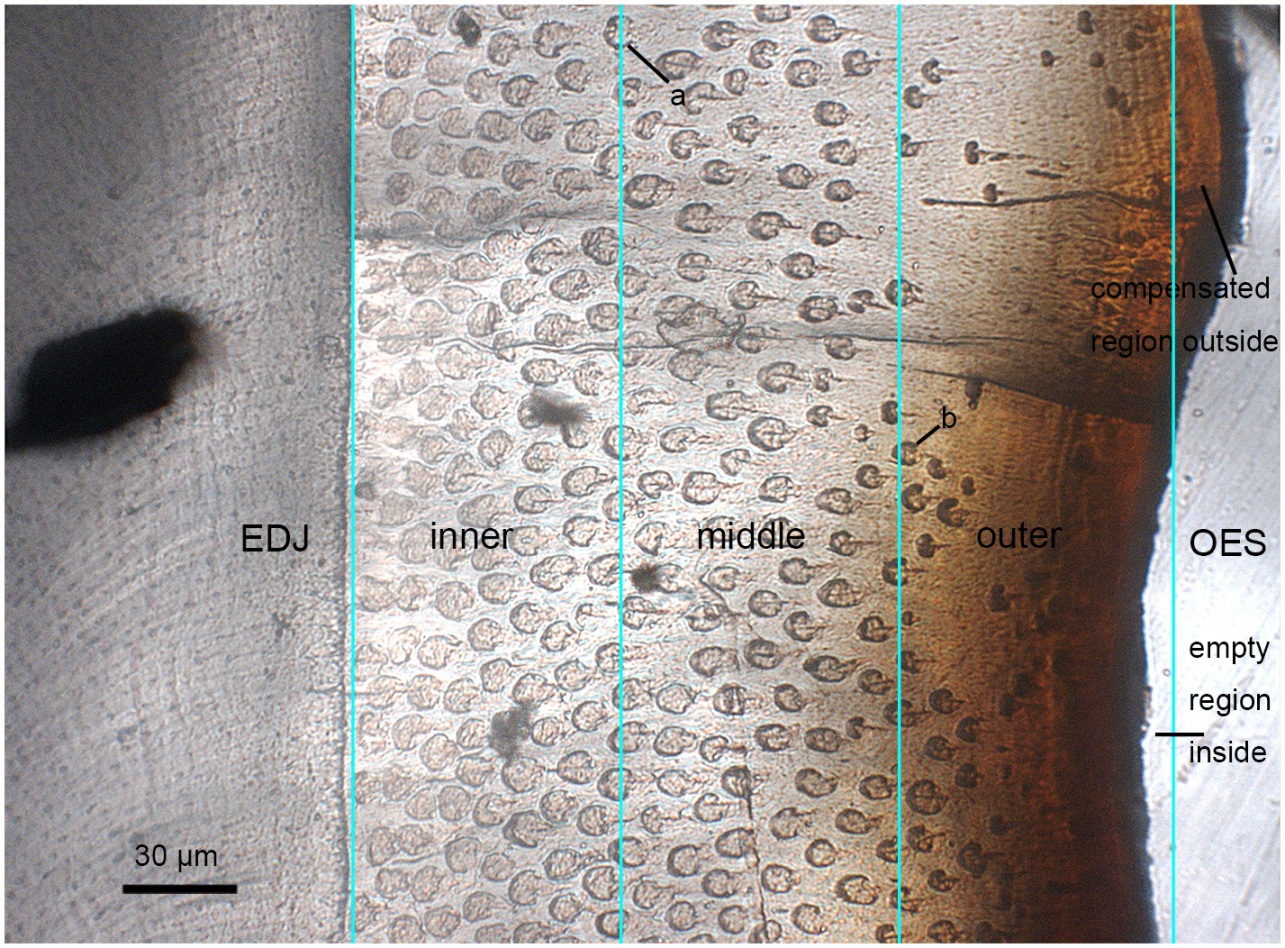
Schematic diagram showing the method used for calculate the prism density. The cross-section of the enamel of left M2 (V 20297.6) is divided into the inner, middle and outer zones with equal width. Because the EDJ and particularly the OES are usually uneven, the boundary is drawn in a way that the empty region inside the line will be roughly compensated by the enamel outside of the line. The area of each zone is equal and the number of the prisms in each zone can be counted. For a prism that is crossed by the line as two roughly equal parts (a), then 0.5 prism will be counted for each zone on the left and right side of the line. If a prism is unevenly divided by the line (b), then the prism will be counted as belonging to the zone in which the larger part of the prism is located.

To quantitatively describe prismatic enamel microstructure in multituberculates and facilitate its practical use in comparison, Fosse [59,60] presented a method to estimate the maximum prism density (MPD) for the enamel of multituberculates in which the prisms are regularly and closely packed (see discussion). In *Lambdopsalis* the prisms are irregularly packed and the true density decreases remarkably from the inner region toward the outer region in cross-section of tooth enamel (Figs 4A, 4B and 6). In order to measure the prism density in a more accurate way and reflect the variation of the density in different enamel zones so that prism density comparison is meaningful among multituberculates, we adopt a different counting method (Fig 6). In the method we evenly divided the enamel between the EDJ and OES into three zones in the enamel cross-section and directly counted the prism number in each zone (Fig 6). These zones are the same as those we used to calculate the average prism diameters except that the three areas are equal and the size of the area is known. Because the EDJ and particularly the OES are usually uneven, to ensure the inner and outer zones are roughly equal to the middle one, we draw the boundary line in a way shown in Fig 6. The numbers of prisms in three zones of different teeth that we examined are presented in the S1 Table. With the prism number in a given area, we can convert the data into the prism density, that is, the number of prisms per mm^2^ in each zone (Table 2).

In order to compare with published data of the MPD from other multituberculates, we also use the same method [27,59] to calculate MPD. However, instead of treating the three zones as a whole, we chose an area with relatively regular prism pattern, usually in the inner zone or in the middle zone, to count the average mutual central distance (d) and calculate the MPD. The number is listed in Table 2.

### 4) Enamel spindles/tubules

Enamel spindles in modern oral histologic study usually refer to short linear defects with a bulbous terminal expansion or an intermittent expansion, arising at the EDJ by following the prism course and extending into the enamel with a length rarely longer than 1/5 of the enamel thickness, and are always found in the cuspal area [61]. There is an analogous structure called enamel tubules [62], or just tubules in early mammals; the difference is that the latter can extend from the EDJ to the OES and is longer than the enamel spindles without limit and regulations [62].

**Fig 7:**
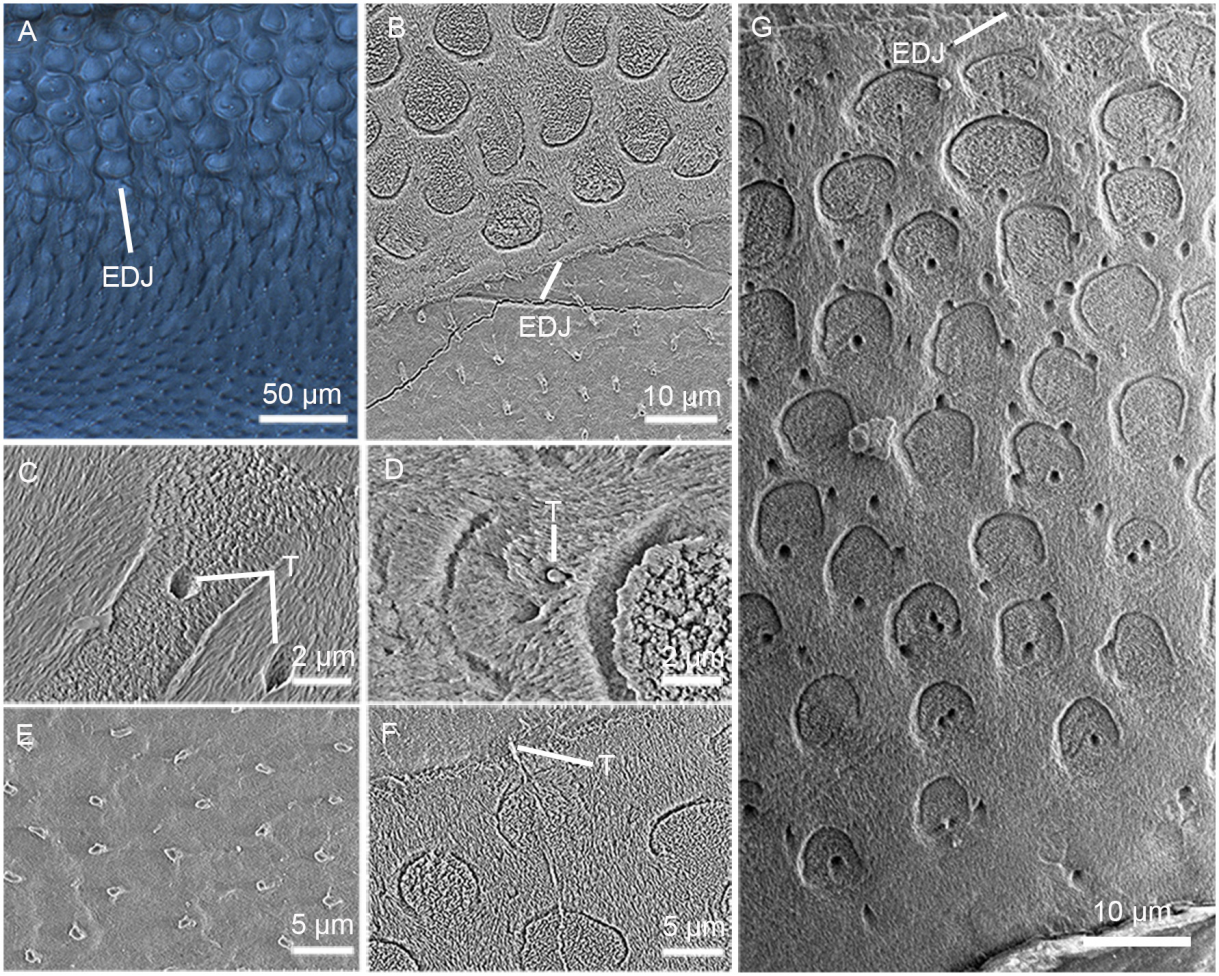
Enamel tubules in the tooth cross-section of *Lambdopsalis bulla*. (A–B) Enamel tubules near the EDJ of left M2 (V 20298.7); (C) Enamel tubules in the enamel of right M2 (V 20298.8) after acid treatment, which are found to be present in the prism cores (right) as well as in interprismatic enamel (left); (D) Enamel tubules still with peritubular dentine in left M2 (V 20298.7) with insufficient acid treatment. (E) Dentinal tubules of left M2 (V 20298.7) with same peritubular dentine like the enamel tubules. (F) Enamel tubule in left M2 (V 20298.7) with a bulbous terminal expansion is perpendicular to the cross-sections of prisms and paths through the enamel layer. (G) Distribution and density variation in the entire enamel of right m2 (V 20297.4) in which enamel tubules are denser in the inner zone and have a random distribution. The tubules have no one to one relation with prisms. Fig 7A is an optic image and the rest are SEM images.

**Fig 8:**
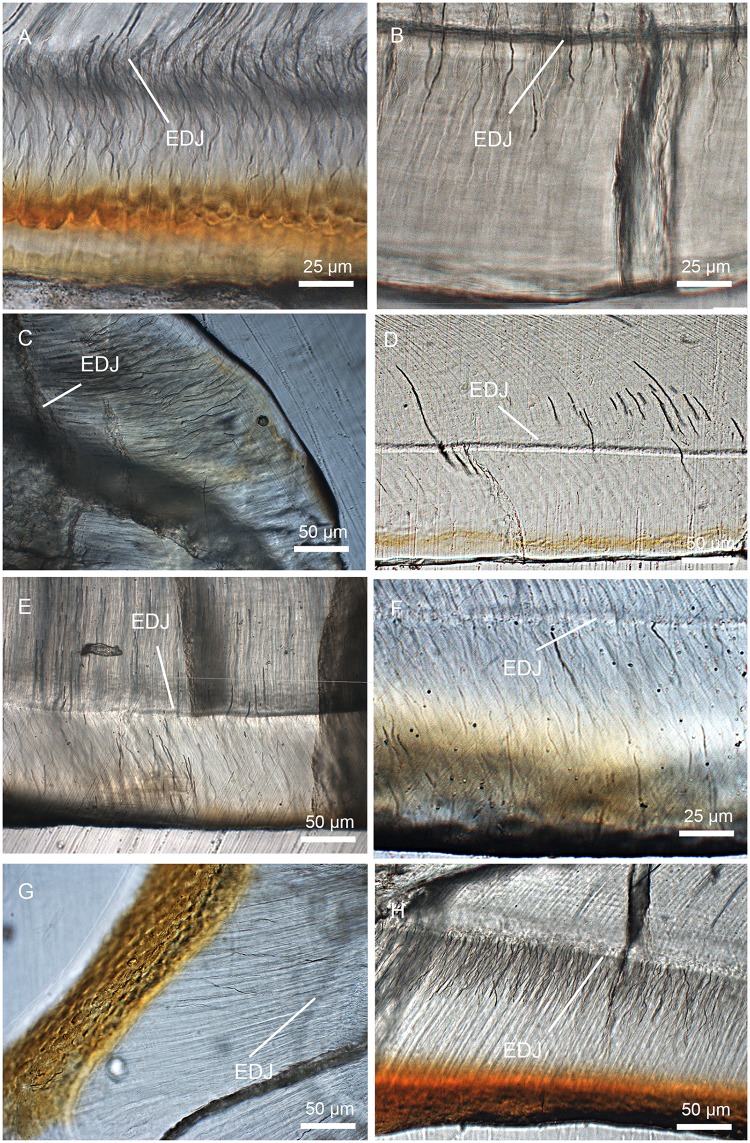
Optic images showing enamel tubules in the longitudinal section of tooth in *Lambdopsalis bulla*. (A) Right i2 (V 20297.3); (B) Right di (V 20300.1); (C) Right P4 (V 20299-3); (D) Right p4 (V 20299-4); (E) Right m1 (V 20297.5); (F) Left M1 (V 20298.6); (G) Right M2 (V 20297.8); (H) Right m2 (V 20297.4). The spiral centrifugal course and branches of enamel tubules are shown in (A). (B) and (F) show that the lengths of enamel tubules can relatively consistent in the same tooth; (D) and (H) show that the tubules can have unstable and inconsistent lengths; (C) and (E) show that the enamel tubules begin in the middle of the enamel layer and extend outward.

Enamel tubules are abundant in the enamel of all teeth of *Lambdopsalis bulla* we examined, which are visible in both SEM and optic images (Figs 7 and 8). In this regard, *Lambdopsalis* enamel differs from those of other multituberculates where enamel tubules are few or even absent [27]. In the cross-sections of *Lambdopsalis* teeth, enamel tubules are found to be present in the prism heads (Fig 7A) as well as in interprismatic enamel (Fig 7B), which appear as small holes in crystallite fabric after acid treatment (Fig 7C), but appear as tubules surrounded by peritubular dentine with insufficient acid treatment (Fig 7D). Enamel tubules are generally regular in size. Most of them are nearly 0.5 μm in diameter although the maximum may reach 1 μm in few cases. The shape, size and structure of enamel tubules are consistent with the dentinal tubules (Fig 7E). A few prisms are perpendicular to the main course of prisms showed in the SEM image in which the bulbous terminations of tubules show clearly (Fig 7F).

Enamel tubules in *Lambdopsalis* have a zigzag or spiral centrifugal course or branches. They are not mutually parallel and only follow the course of prisms over a short distance. In contrast, prisms are usually straight and mutually parallel each other in most area of the enamel (Figs 7G and 8). Unlike many other mammals in which the tubules are found in the inner enamel [62], the enamel tubules of *Lambdopsalis* are also present in the cuspal and the lateral enamel. The tubules have unstable and inconsistent lengths. Most of them arise from the EDJ, with some extending to the surface of enamel (Fig 8A, 8C and 8G), whereas some are apparently continuous extensions of the dentinal tubules and usually end in the middle part of the enamel layer (Fig 8B, 8D and 8F). Some tubules begin in the middle of the enamel layer and extend outward (Fig 8E).

### 5) Prism packing in the cross section

**Fig 9:**
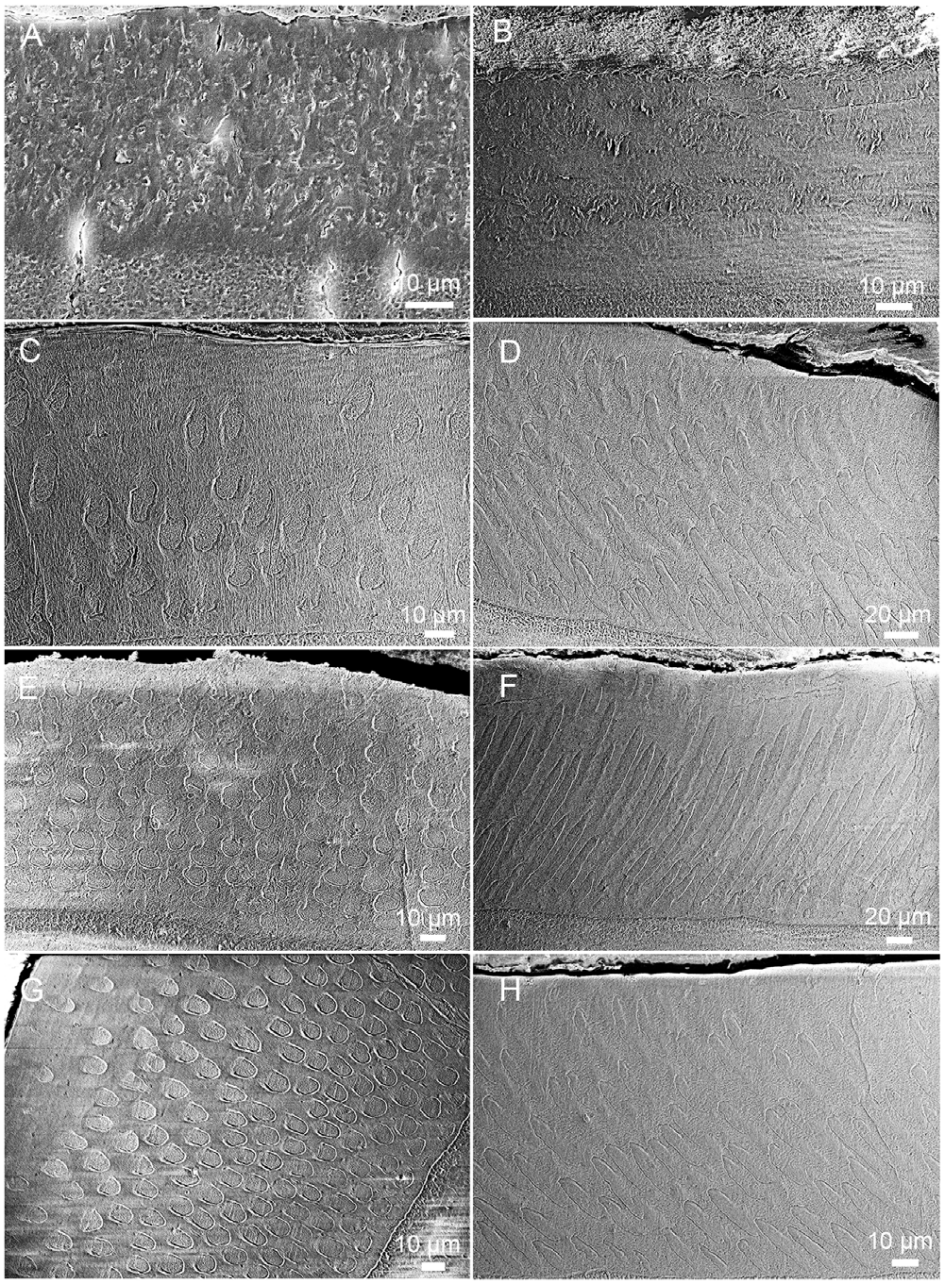
SEM images showing enamel type in enamel of incisors in *Lambdopsalis bulla*. (A–B) Right DI (V 20299-1); (C) Right di (V 20299-5); (D) Left di (V 20301.1); (E) Right I2 (V 20298.2); (F) Right I2 (V 20298.3); (G–H) Right i2 (V 20300.2). The images on the left are cross-sections and those on the right are the longitudinal sections.

**Fig 10:**
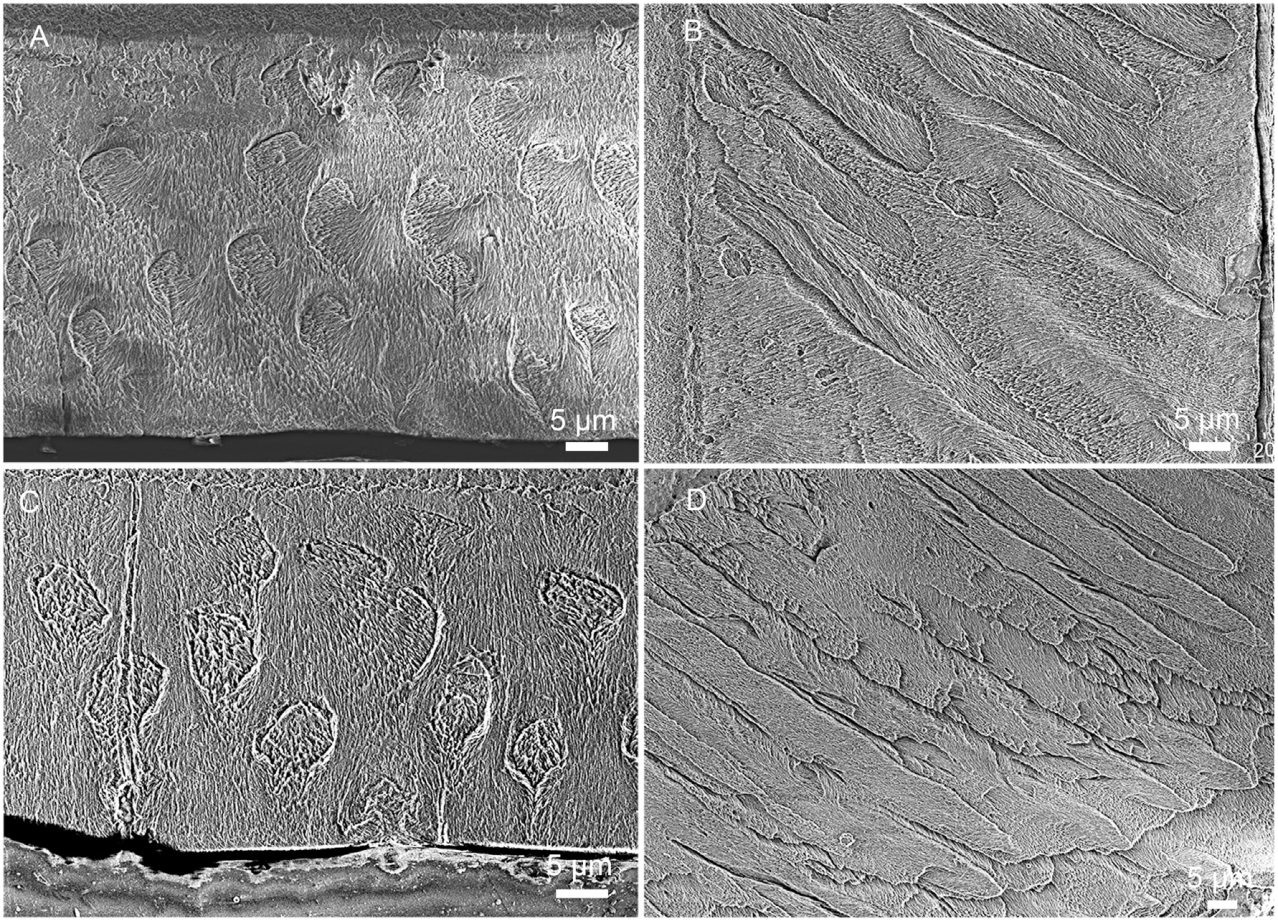
SEM images showing enamel type in enamel of premolars in *Lambdopsalis bulla*. (A–B) Right P4 (V 20299-3); (C–D) Right p4 (V 20299-4). The images on the left are cross-sections and those on the right are the longitudinal sections.

**Fig 11:**
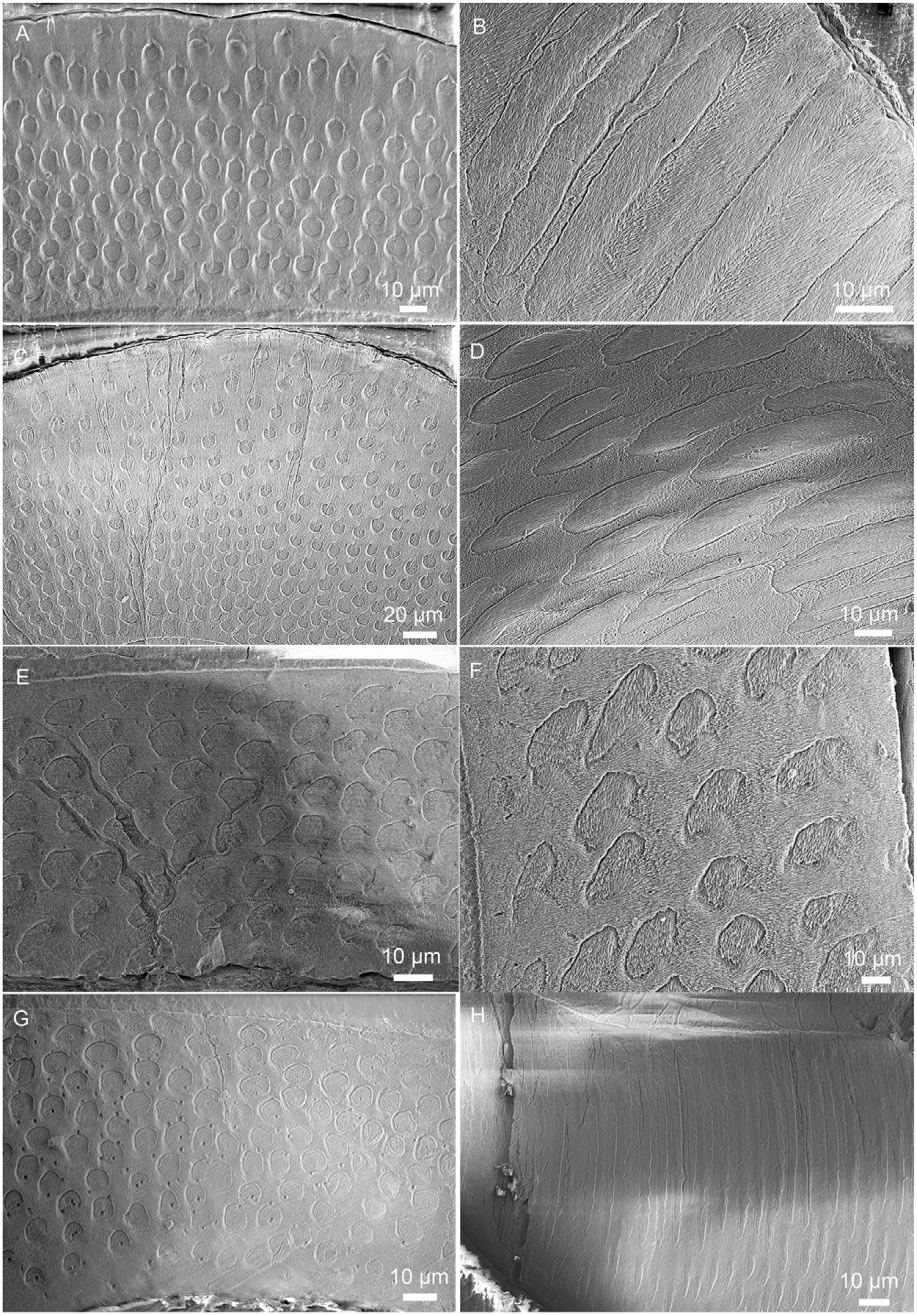
SEM images showing enamel type in enamel of molars in *Lambdopsalis bulla*. (A) Left M1 (V 20298.5); (B) Right M1 (V 20298.6); (C) Left M2 (V 20298.7); (D) Right M2 (V 20298.8); (E–F) Right m1 (V 20297.4); (G–H) Right m2 (V 20297.4). The images on the left are cross-sections and those on the right are the longitudinal sections.

The two-dimensional arrangement of prisms in the cross section is referred here as the prism packing [34]. In cross sections of all teeth of *Lambdopsalis*, most prisms have the open end of sheaths facing the OES (Fig 4A and 4B), a pattern that is consistent in all teeth observed. However, the arrangement of prisms is erratic and shows no regular pattern. Some layers with organization of prisms in horizontal rows with alternating positions that conform to pattern 3 of Boyde’s system [54] (Figs 9G, 10A, 11E and 11G), whereas prisms in other areas are arranged in approximately vertical rows (Fig 11A and 11C) and fit to pattern 2 [54]. Thus, we echo the opinion of Carlson and Krause [27] that the prism type of Boyde’s system is difficult to be used in the study of multituberculate teeth.

### Enamel types

In general, with the exception of the upper deciduous incisor (V 20299–1, Fig 9A), the tooth enamel of *Lambdopsalis bulla* presents, in cross-sectional view, an outer layer with parallel crystallite enamel and the inner and middle zones with radial packing prisms. Enamel prisms in the inner and middle zones tend to have the open end of sheaths facing the OES (Figs 9C, 9E, 9G, 10A, 10C, 11A, 11C, 11E and 11G). In most cases the prisms has linear arrangement, although some are vertical to the EDJ and OES (Fig 11A and 11C), whereas others incline to the EDJ at an angle (Figs 9G, 10A, 10C, 11E and 11G). In other cases, the arrangement of the prisms does not seem to have any regular pattern (Fig 9C and 9E).

In the longitudinal section of tooth enamel, prisms are parallel to each other and have an angle to the EDJ; they diverge toward apex of the tooth from EDJ to OES in the lateral enamel of cheek teeth. The angle is approximately 45° at the cervical area of tooth toward the root (Figs 9D, 10B and 11B), and increases from the cervical area of the tooth crown to the EDJ at some cuspal area where it becomes perpendicular to the EDJ (Figs 9F, 10D and 11H). There are some variations of the prism orientation in various parts of the cuspal and lateral enamel (Figs 9H and 11F). In general, the same orientation pattern retains in nearly all teeth that we examined. The enamel type of such a prismatic layer is usually classified as the radial enamel [34], which differs from other enamel types such as the tangential enamel, Hunter-Schreger bands, and irregular decussation in which the prisms are not parallel to each other but decussated in different ways.

**Fig 12:**
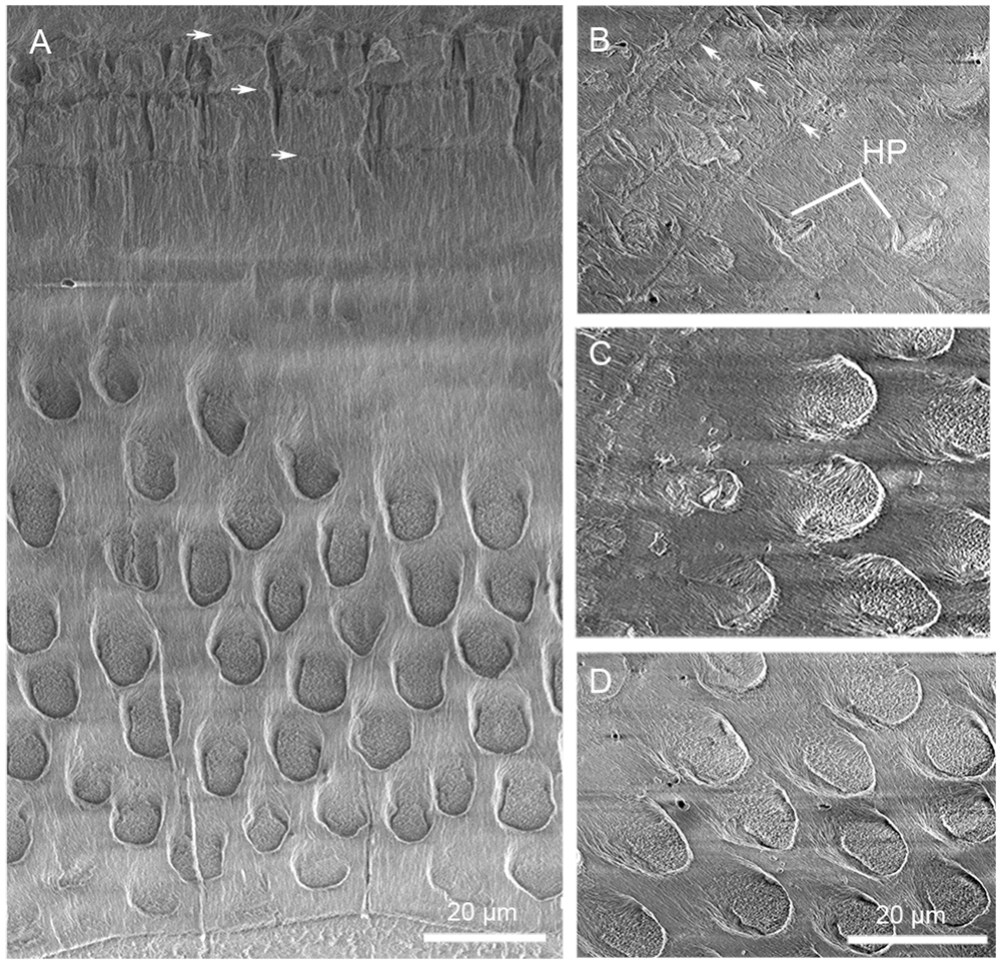
SEM images showing enamel type in the cross-section of right i2 (V 20300.2) in *Lambdopsalis bulla*. (A) Enamel type changing from the EDJ (bottom) to the OES (top); (B–D) Details showing enamel type of outside (B), middle (C) and inside (D). B–D with the same magnification. Incremental lines are marked by a series of white arrows. Abbreviations: HP: hypogenetic prism.

Aprismatic enamel is also common and usually found near the OES of *Lambdopsalis*. The aprismatic layer in deciduous teeth is more distinctive than those in permanent teeth (Fig 4D and 4E). The thickness of the aprismatic layer varies irregularly even in the same tooth (Fig 6), in different permanent teeth (Figs 9–11), and between deciduous and permanent teeth (Figs 4, 5 and 9). Usually, the boundary of the aprismatic and prismatic layers is not even (Fig 12) and the prisms change step by step from the middle portion to the OES (Fig 12A–12C). There are some incremental lines parallel to the aprismatic layer (Fig 12A) and some small and hypogenetic prisms are occasionally found in the outer enamel (Fig 12B).

**Fig 13:**
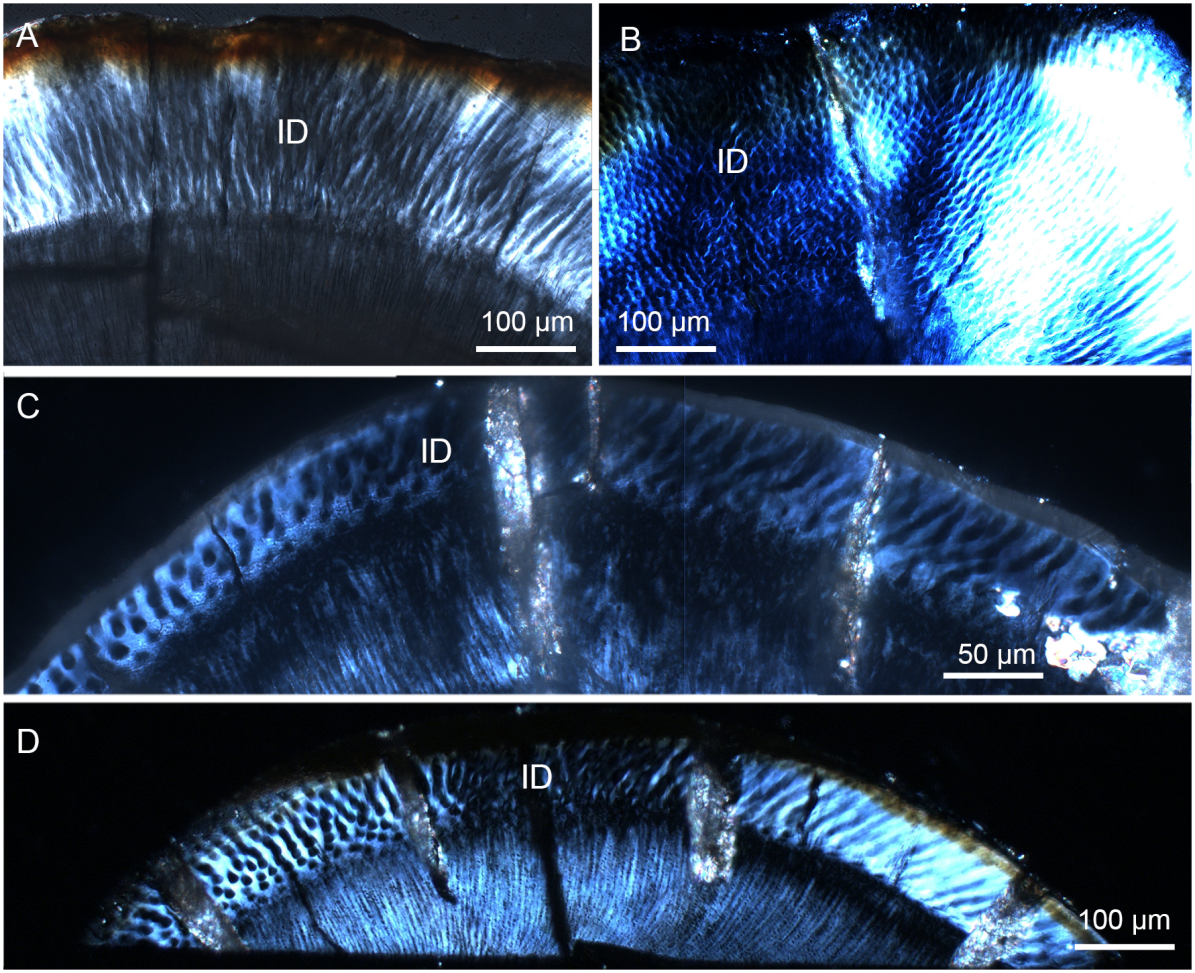
Polarized light images showing irregular decussation in the cross-section of the tooth in *Lambdopsalis bulla*. (A) Right i2 (V 20300.2); (B) Left M2 (V 20298.7); (C) Right P4 (V 20299-3); (D) Right I2 (V 20299-2). ID: irregular decussation.

**Fig 14:**
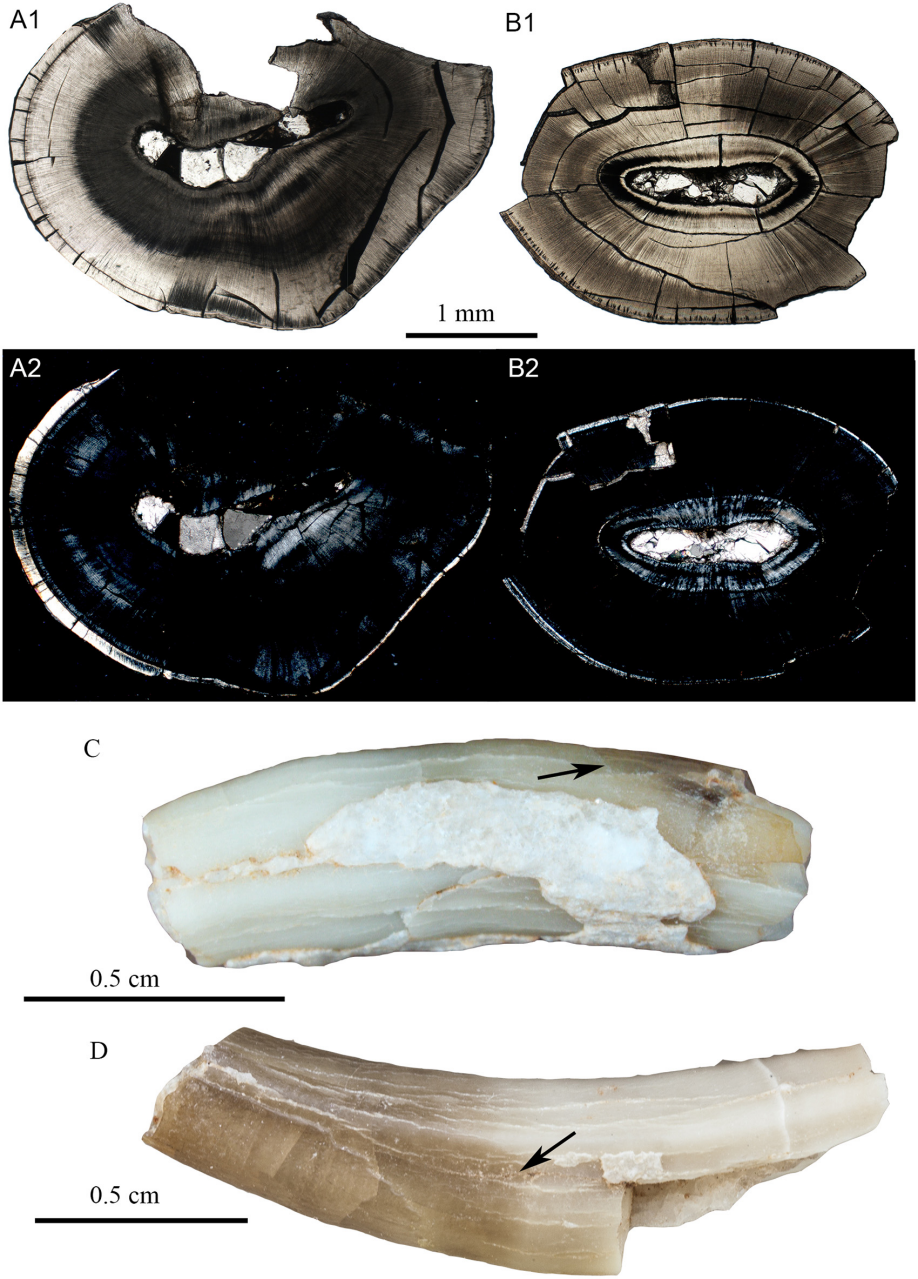
Schmelzmuster of the deciduous incisor in *Lambdopsalis bulla*. (A1-2) Optic and polarized light images of the cross sectional views of the right lower deciduous insisor (V 20300.1); (B1-2) Optic and polarized light images of the cross sectional views of the right upper deciduous incisor (V 20299-1); (C) Right upper deciduous incisor (V 20715.1) and (D) Right lower deciduous incisor (V 20300.1) showing the distribution of the enamel on the teeth. The arrow marks the distal ending of the enamel. A and B are composed from images of partial views of the cross section.

**Fig 15:**
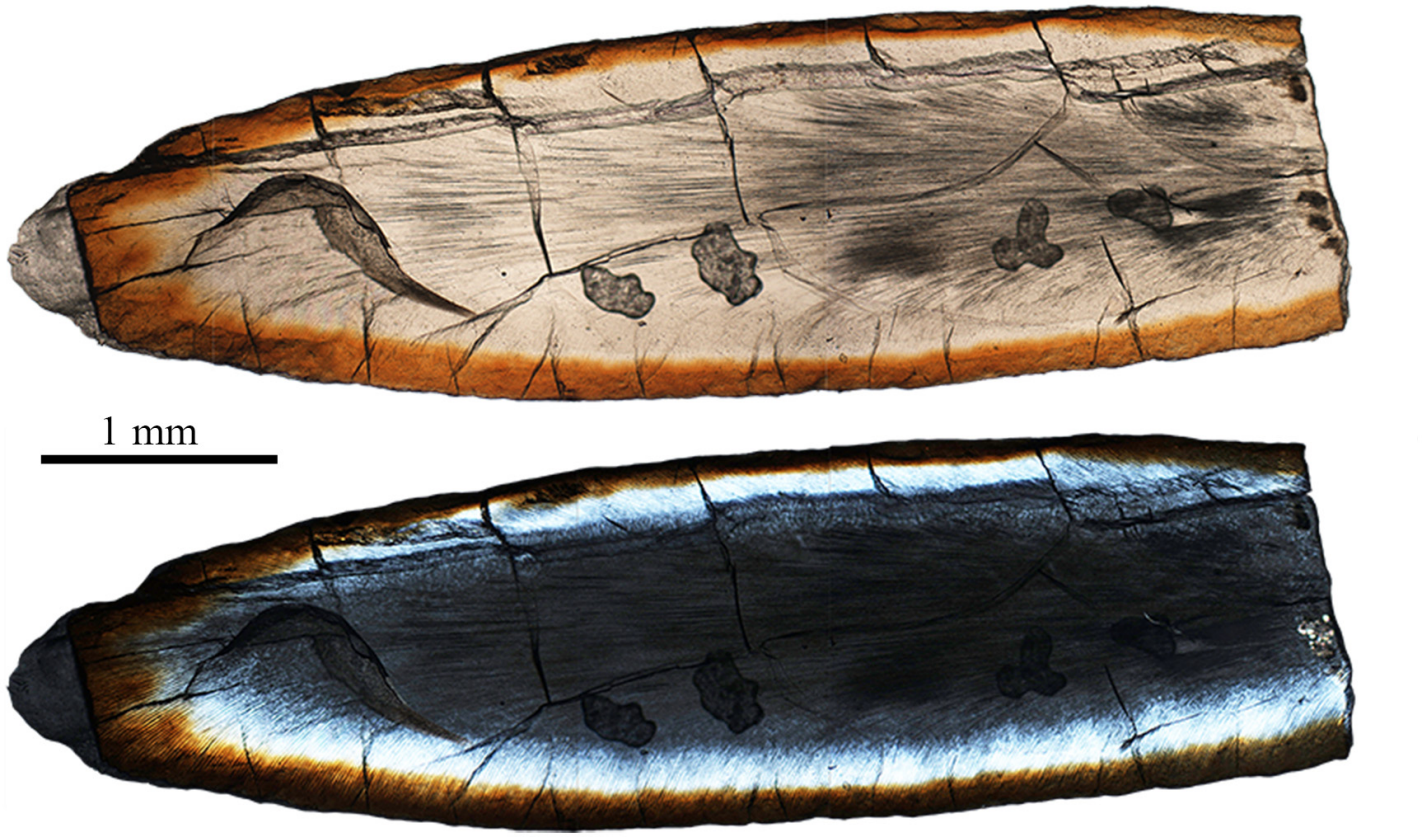
Schmelzmuster of the left I2 (V 20298.1) of *Lambdopsalis bulla*. Optic image (upper) and polarized light image (lower) through the longitudinal section. The images are composed from several images showing partial section.

**Fig 16:**
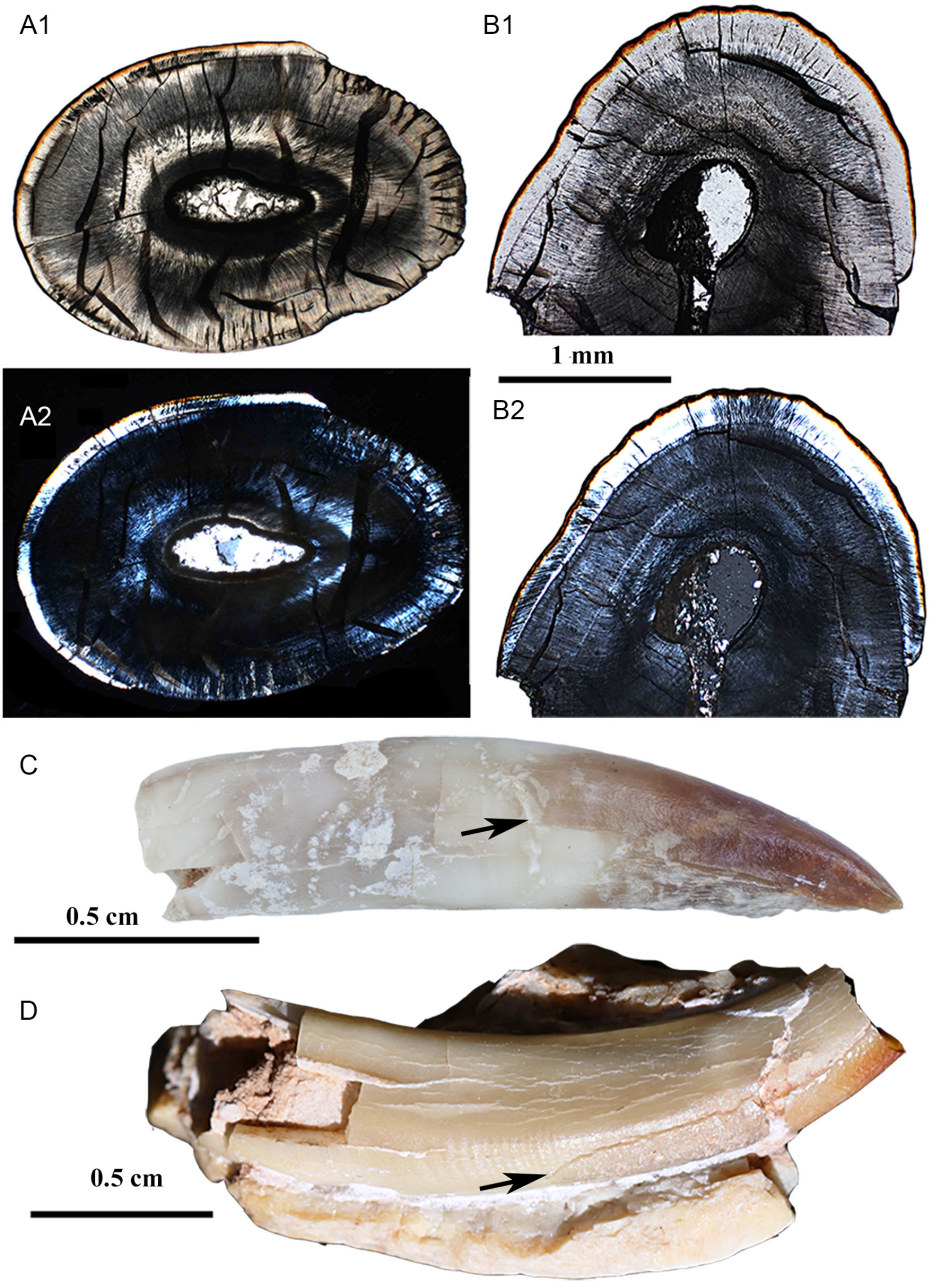
Schmelzmuster of the incisors in *Lambdopsalis bulla*. (A1-2) Optic and polarized light images of the cross sectional views of the right I2 (V 20298.2); (B1-2) Optic and polarized light images of the cross sectional views of the right i2 (V 20300.1); (C) Right I2 (V 20298.2), and (D) Left lower i2 (V 20716.1) showing the positional relationship of the distal edge of the enamel with the margin of alveolus. These teeth are not from the same individual.

**Fig 17:**
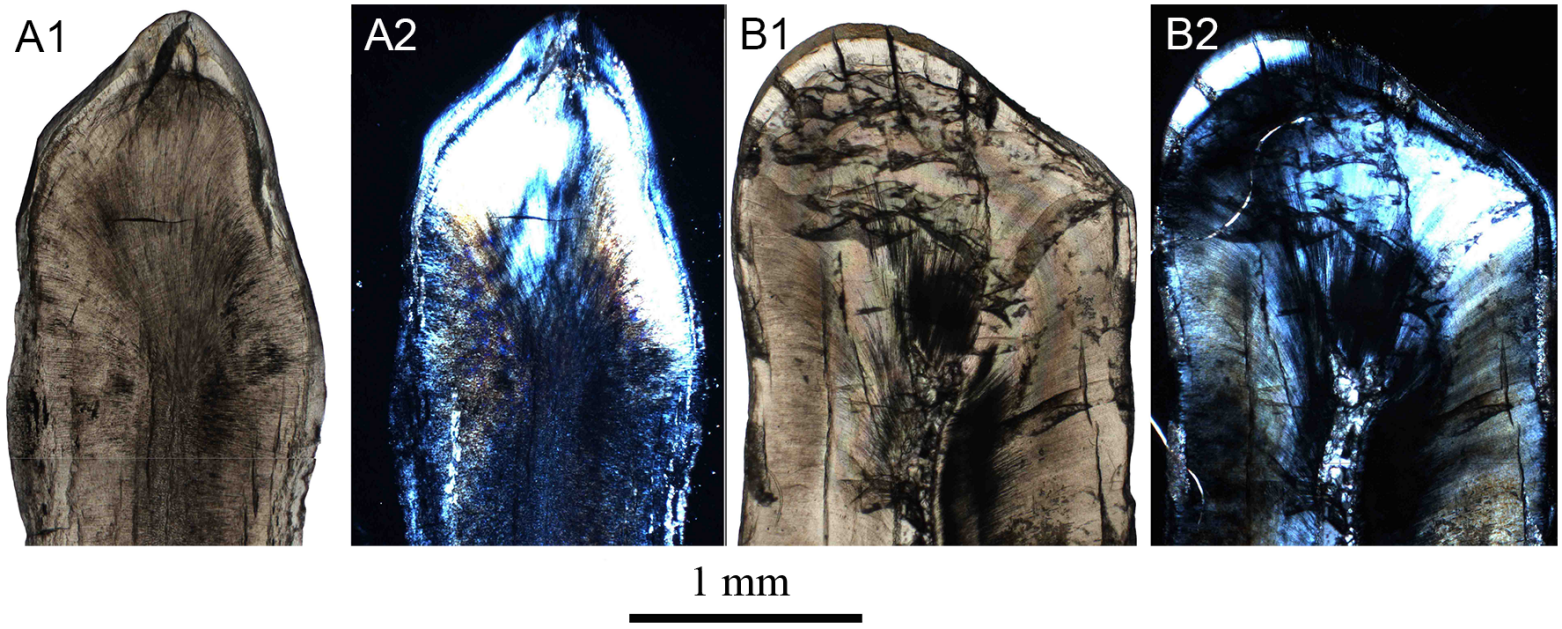
Schmelzmuster of the premolar in *Lambdopsalis bulla*. (A) Right P4 (V 20299-3); (B) Right p4 (V 20299-4). 1. Optic images; 2. Polarized light images. The two teeth are from the same individual.

In addition to the radial enamel and aprismatic enamel, there are some prisms that change the orientation in the basin or the cuspal peak of the tooth. Irregular decussation of prisms appears to exist in the mesiobuccal side of the narrow enamel bands within a very small space on both upper and lower incisors of *Lambdopsalis* (Fig 13A and 13D), in the cuspal area of premolars (Fig 13C), and in the valley of molars (Fig 13B). It seems that the enamel in areas of the tooth that have a great degree of curvature, the prisms are decussated irregularly, although usually in limited areas. The thickness of the aprismatic enamel also gradually decreases from the tooth tip toward the root along with the reduction of enamel thickness (Figs 14–17).

### Schmelzmuster

In most teeth, several enamel types are combined in a very distinct spatial arrangement. This three-dimensional arrangement of enamel types in one tooth is defined as the Schmelzmuster [63]. The Schmelzmuster in all teeth of *Lambdopsalis bulla* we examined seems to be simple. The most common Schmelzmuster is the combination of radial enamel in the inner region of the enamel and aprismatic enamel in the outer layer (Figs 9–11). As discussed above, irregular decussations can be found in parts of permanent teeth where the tooth has a great degree of curvature (Fig 13). The percentage of each kind of enamel type varies on different parts of a tooth. Generally, the radial enamel increases toward the cuspal area; the proportion of the aprismatic enamel becomes greater toward the cervical area. The irregular decussation only occurs in the enamel with high tortuosity of gross morphology, such as the mesiobuccal side of incisor and the valley of the molars.

### Dentition types

Dentition types are the variation in Schmelzmuster throughout the dentition [36]. As discussed above, there are few changes of the Schmelzmuster in any tooth of *Lambdopsalis bulla*.

In the lower deciduous incisor (V 20300.1), radial enamel was only found in the anterior portion of the tooth, in which prisms are few (Fig 2C). The enamel of the mesiobuccal part of lower deciduous incisor is the thickest with more prisms than other parts of the tooth; the enamel gradually thins toward the medial, lateral and distal sides of the tooth and the enamel type changes from the radial to aprismatic (Fig 14A). The enamel of the upper deciduous incisor also gradually thins toward the medial, lateral and distal sides (V 20299-1, Fig 14B). The enamel of the upper deciduous incisor is aprismatic, as described above, but differs from that of the outer aprismatic layer of permanent teeth in having crystallites arranged in columns (Fig 5A and 5B). The enamel of the upper deciduous incisor is the thinnest among all teeth we examined.

In both upper (V 20298.2) and lower permanent incisor (V 20300.1), the enamel forms a band covering the buccal side for the most part of the tooth body (Figs 15 and 16). The enamel of the lower incisor is at least twice the thickness of the upper one. On the anterior portion of the nearly unworn incisor (V 20298.1), the enamel also extends to the lingual side to a considerable distance. The incisor has the radial enamel with longest prisms in the tip where the enamel is the thickest (Fig 15). In the mesiobuccal area of the upper and lower incisors, the enamel shows some local irregular decussation (Fig 15). The orientation of the prisms in the cervical part is oblique to the root and from the lateral side to the mesiobuccal area the oblique angle of prisms gradually changes to nearly vertical to the EDJ. The enamel disappears on the unerupted portion of the deciduous and permanent incisors at the point not far from the margin of the alveolus (Figs 14C, 14D, 16C and 16D).

The gross shape of the premolars in *Lambdopsalis* is somewhat incisor-form and strongly reduced in proportion to enlarged multicusped molars [64]. The enamel microstructure of the premolars is similar to that of the incisors where the radial enamel is predominant, the irregular decussation exists in the mesiobuccal of the tooth, and the aprismatic enamel occurs near the root. Unlike the incisors, the thickness is similar in the upper (V 20299-3) and lower premolars (V 20299-4) and the enamel structures are similar to that of the permanent lower incisor (Figs 16 and 17).

The molars have relatively complex shapes and usually have a complex three-dimensional pattern of prisms within the enamel. However, except for some irregular decussation occurring in the valley (Fig 13B), the enamel type is relatively simple in molars of *Lambdopsalis*, as in other teeth. The prism size, prism density, and the distribution of aprismatic enamel vary among enamels of different cusps and in different parts of the same cusp; these variations appear related to the thickness of the enamel and the topographic features, such as the crest cusps and the central valley.

**Fig 18:**
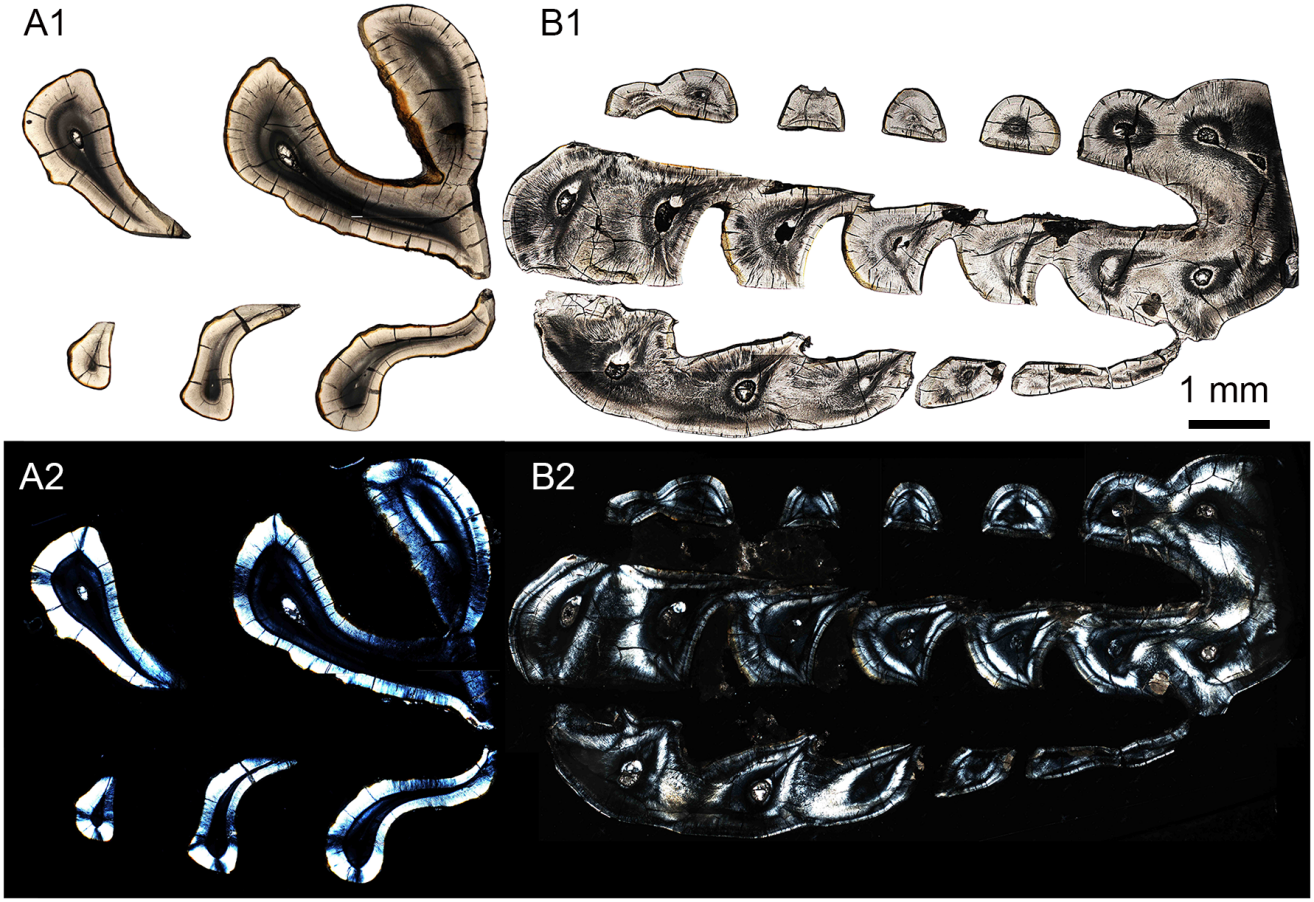
Schmelzmuster of the upper molar in *Lambdopsalis bulla*. (A) Left M2 (V 20298.7); (B) Left M1 (V 20298.5); 1. Optic images; 2. Polarized light images. All pictures through the transverse section. The two teeth are not from the same individual.

**Fig 19:**
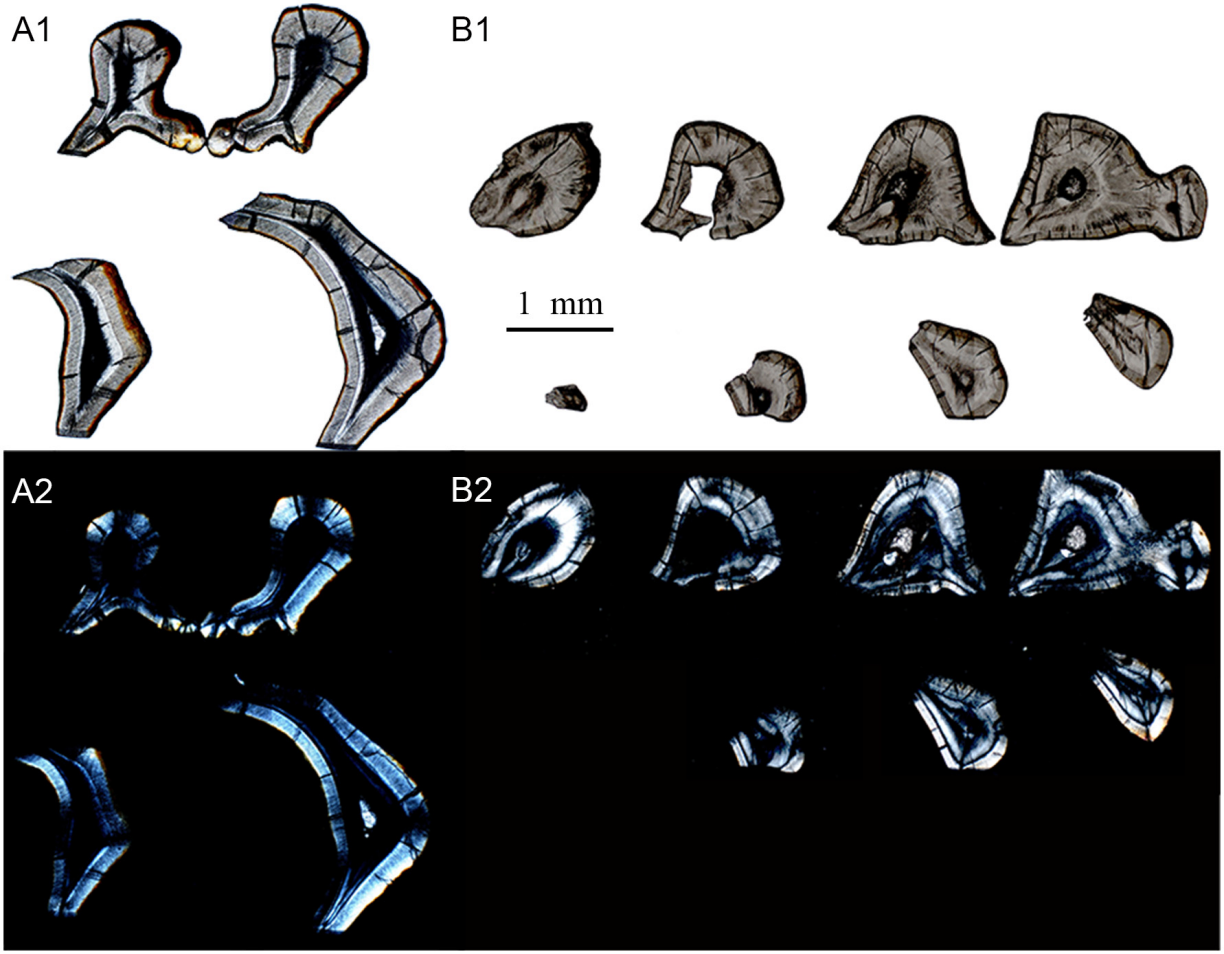
Schmelzmuster of the lower molars in *Lambdopsalis bulla*. (A) Right m2 (V 20300.3); (B) Right m1 (V 20298.4); 1. Optic images; 2. Polarized light images. All pictures through the transverse section. The two teeth are not from the same individual.

There are a few general trends of the enamel variation in the molar of *Lambdopsalis*. The enamel thickness of the second molar (V 20298.5 and V 20300.3) is usually thicker than that of the first molar (V 20298.4 and V 20298.7) in corresponding areas (Figs 18 and 19). The enamel thickness of the distal side of the tooth cusp is usually thicker than that of the mesial side in the upper molars (Fig 18), but the condition is reversed in the lower molars (Fig 19). The enamel of the central valley is significantly thinner than that in the lateral side of the same cusp in most teeth, except that the enamel of the cusps in the medial row of M1 has equal thickness in both sides. The average prism size in the thicker enamel is smaller than that in the thinner enamel and the IPM in the thicker enamel occupies more space than that in the thinner enamel. The aprismatic enamel is thicker in the outer zone of the thicker enamel in which there are some hypogenetic prisms and small prisms.

It is commonly seen that the second upper and lower molars and the permanent incisors of *Lambdopsalis* have distinctive reddish pigmentation on the crown surface but the enamel of the other teeth is in relatively light color (Figs 15, 16, 18A and 19A), as noted by Miao [64]. This condition is also present in *Sphenopsalis nobilis*, a species that is now in the same family of Lambdopsalidae [65]. The pigmentation is likely similar to those in teeth of some rodent and insectivore, which may be caused by iron and interpreted as to enhance the mechanical and acid resistance strength of permanent teeth [66–71]. The differential pattern of the pigmentation in the dentition of *Lambdopsalis* was believed to be natural, which may be related to the physical (enamel structure) and/or chemical differences of the enamel in the dentition. Our observation shows that a very thin reddish layer is also present in premolars (Fig 8C and 8D) and the first molars (Fig 8E and 8F), which is less distinctive than that of M2/m2 (Fig 8G and 8H) and permanent incisors (Figs 15 and 16). The only tooth we did not find any evidence of pigmentation is the deciduous incisor (Figs 8B and 14A). The pigmented enamel in the second molars and permanent incisors is thicker than that of the first molars and the thickness varies irregularly in different parts of the same tooth (Figs 4A, 4C, 6 and 15). In any tooth, however, the thickest reddish enamel is less than a half of the total thickness of the entire tooth enamel. The enamel microstructures of the pigmented portion and the unpigmented part in the permanent teeth have no significant difference (Fig 15). The pigmented zone is not limited in the aprismatic layers; it also extends to the prismatic layer or to the transitional region (Fig 6). There is no sharp boundary between the pigmented and non-pigmented zones in *Lambdopsalis*.

## Discussion

### Prism size

Fosse et al. [36] proposed that the prism size have diagnostic value for the taxonomy of multituberculates. Carlson and Krause [27] studied the distribution of prismatic enamel in 30 Late Cretaceous and early Tertiary multituberculate genera and defined two types of multituberculate prisms according to the average prism diameter and prism shape: small and circular prism (average diameter = 3.6 μm) versus large and arcade-shaped (average diameter = 8.2 μm), although the prism diameter in conspecific individuals in their samples displayed a variation by up to 3.0 μm. They supported the conclusion of Fosse et al. [36] that the “gigantoprism” characterizes the Taeniolabidoidea and the small prism characterizes Ptilodontoidea. In the multituberculate phylogeny analysis of Kielan-Jaworowska and Hurum [72], the small prismatic enamel had been coded for most post-plagiaulacoid multituberculates except for Ptilodontoidea. Fosse et al. [73] noted yet another type of multituberculate enamel, SCE, in the enamel of Plagiaulacoidea. Wood and Stern [52] and Wood and Rougier [33] also defined the plesiomorphic prismatic enamel (PPE) for the small, arc-shaped and irregular packed prisms with seam. The origins of these prism types in multituberculates are still controversial [33,72,74–78]. In any case, the prism shape and size can prove instrumental in reflecting the relationships among primitive multituberculate taxa [77], as exemplified in the case of *Argentodites* [22].

The average prism diameter of *Lambdopsalis bulla* in our samples is 8.33 μm, which by definition belongs to the gigantoprism enamel and again confirmed the conclusion of Fosse et al. [36] and Carlson and Krause [27] to place *Lambdopsalis* in Taeniolabidoidea. However, the enamel of *Lambdopsalis* shows that the prism size, as represented by its diameter, is not consistent. The largest prism in our sample has a diameter about 13.68 μm while the smallest is 4.25 μm. The prism diameter in these conspecific individuals displayed a size variation by up to 9.4 μm. The standard deviation about the prism diameter in a single tooth is as high as 1.63 and that of the whole sample is up to 1.81. The prism diameter usually decreases from the EDJ to OES (Table 2) and from the crown toward the cervical part in the same tooth. The smallest prism diameter falls into the size range of the small prism common in ptilodontoids, whereas the large prisms are typical of gigantoprisms (S1 Table). A cross section of a specimen may reveal prisms with large diameters near the EDJ and small prisms toward the OES (Fig 6). The aprismatic enamel is quite thick in the enamel of *Lambdopsalis*, which may reach up to one-third or even half of the entire enamel thickness and the small prisms often occur near the aprismatic area. Thus, inappropriate sampling may result in significantly different prism diameters for the same tooth. The diameter of prisms, at least in *Lambdopsalis*, is sensitive with the depth of enamel examined. Prisms in the middle zone of the enamel are most stable in diameters and shape. Our examination of the *Lambdopsalis* enamel shows that once the section is cut sufficiently deep, as did in Carlson and Krause [27], the estimate of the prism size for a species should be reliable.

Our observation shows that variation of prism diameter also exists among teeth, but not so dramatically. In general, the prism size of molars is larger than that of the premolars and the lower incisor, but is similar to that of the upper incisor and deciduous lower incisor (Table 2). Nonetheless, the average diameters in all teeth fall in the range of gigantoprisms, which suggests that the middle zone of the enamel from any tooth of a species in multituberculates would present a consistent prism size estimate, either the small (normal) prisms or gigantoprism.

### Prism shape

The prismatic enamel is generally considered as those characterized by having bundles of similarly oriented crystallites that are separated from other prisms by a prism sheath and by interprismatic crystallites [44,79], regardless the shape of the sheath [54] (it could be circular or in arch shape). Fosse et al. [36] and Carlson and Krause [27] considered that the prism size of multituberculates is consistently corresponded with their shapes such that the small prism and closed, circular sheath are generally typical for Ptilodontoidea and the large “gigantoprism” and arch-shaped sheath are characteristic for other post-plagiaulacoid multituberculates.

However, prism shapes vary even within the same tooth. Carlson and Krause [27] and Krause and Carlson [75] illustrated that in the tooth of *Mesodma*, a Late Cretaceous-Paleogene ptilodontoid multituberculate, both circular and arcade-shaped small prisms are present in the same enamel section specimen, with the circular prisms being predominated. The mixed prisms were found adjacent to the EDJ so that their positions are much deeper (toward the EDJ) than in most other sections examined [27]. In *Lambdopsalis bulla*, most prisms are arcade-shaped with the prism sheath nearly three-quarters of a circle. However the prism sheath exhibits some changes from a compressed shape near the EDJ to elongated one near the OES. As a consequence, the open area of the prism sheath decreases in size or even diminishes from the EDJ to the OES so that some sheaths are even closed in the tail part to display a circular pattern (Fig 6). This observation supports the view that the variation in prism cross-sections can be found at different levels within the enamel in different areas of the postcanine dentition, even within simple enamel types [34]; thus, only the predominate prism type should be considered to bear the phylogenetic information. If one has to code the prism shape as an enamel character, *Mesodma* should be coded as having the circular prism instead of a mixed prism shape.

In contrast to the case of *Mesodma*, in which the circular small prisms are predominant near the EDJ, the microcosmodont *Microcosmodon* uniquely possesses enamel with both circular and arcade-shaped small prisms, with the latter being predominated. More importantly, the mixed prisms are found in all sections of *Microcosmodon* [27,75], which means that the mix prisms are not a local phenomenon and is not sensitive to the depth of the enamel layer observed. Carlson and Krause [27] thought that the *Microcosmodon* condition cannot equate to the coexistence of different prism type in specific area occasionally found in more derived mammals [80,81]. In this case, the mixed prism shape should be coded for *Microcosmodon*.

### Prism density

Fosse [59,60] presented an estimate of prism density (number of prisms per mm^2^) that had been wildly used in quantization of the prisms in multituberculates [14,27,36,59,60,73,75]. In his method, Fosse [59,60] assumed that the prisms are regularly and closely apposed in the cross sections in planoparallel with the ameloblast/enamel contact surface. He used the average mutual central distance (d), which is derived from the true length of mean triangle side of three adjacent prisms, to estimate the maximum number of prisms per mm^2^. The equation to estimate the maximum prism density (MPD) is: MPD = (2×10^6^)/(d^2^)(3^1/2^) [59]. This method is useful to compare enamels that have a relatively even distribution of prisms. It was noted, however, that the estimate of MPD could yield values that are slightly altered [27] and cannot be easily measured in enamels with an irregular prism pattern [60].

As we show in the description, for all permanent teeth of *Lambdopsalis*, except for the upper premolar, the prism density of the inner and middle zones is more or less similar but notably greater than that of the outer zone (Table 2). The only upper premolar we examined is a newly erupted tooth that bears no wear. Its enamel is very thin and the area we could get is very limited. Thus the cross section of this tooth contains only a few prisms that it is difficult to divide it into three zones as we did for other teeth. When its cross-section was eventually divided into three zones in the imaged region (Fig 10A), only 15 prisms were present in the inner zone whose diameters can be measured (Table 2), whereas the prism number in the middle zone can be up to 20. This unusual condition (thin enamel with few prisms) is probably because P4 in taeniolabidoids is no longer used for primary function of mastication [82,83].

The maximum prism density (MPD) of *Lambdopsalis*, which is calculated based on the mutual central distance between prisms [59], shows a different pattern. The molars have a greater MPD than other teeth and the MPD of M1 is the greatest. However, we want to emphasize again that the mutual central distance between prisms in the same cross section of different teeth varies considerably. For instance, the standard deviation of the mutual central distance between prisms was up to 3.91 in the lower deciduous incisor. Thus, the MPD from different teeth should be viewed with caution.

Comparing to the prism density data with other published multituberculates, the MPDs of the permanent teeth (incisors and molars) of *Lambdopsalis* are approximately equal to those of *Taeniolabis taoensis* and *Catopsalis joyneri* [27], which are the lowest in Taeniolabidoidea as well as in cimolodontans.

### Prism seams

The prism seam is a common feature in the enamel of Mesozoic mammals [34,48,49,52,74,84–86]. Its development was interpreted being related to a central groove on the sloping floor-wall of the Tomes' process pit [87]. This feature was further considered to be the restricted plane of crystallite convergence behind and left by the keel developed on the terminal face of the Tomes’ process of the prism [45]. The reduction of the Tomes’ process at the end of the secretory phase, however, is sometimes gradual, leading to the persistence of the seam in enamel consisting otherwise of parallel crystallites [49].

Because of its common occurrence of in early mammals and in all earliest representatives of extant subclasses, the seam was considered probably a plesiomorphic enamel feature in mammals [49,84]. This means that the prismatic enamel without the seam would represent a derived condition, regardless of other attributes of the enamel at the prism level or higher levels of an organization [52]. The evolution of mammalian enamel with or without the seam is complex [33,45,52], which is a subject beyond the scope of this study. However, our study does provide useful data to test some previous hypotheses about the enamel evolution in mammals. For instance, when entertaining the possibility of multiple versus single origination of mammalian enamel prisms, Sander [45] used non-seam enamel with gigantoprismatic prisms in taeniolabidoid multituberculates and non-seam enamel of monotremes as examples to question the convergent evolutionary loss of prisms and the enamel seam as a synapomorphy of synapsids. In a previous study on the enamel of *Lambdopsalis* ([27]: Fig 15B), the enamel prisms did not seem to display the seams in the image. The authors did not discuss whether or not the seam is present in *Lambdopsalis*. As we have shown in this study, *Lambdopsalis*, an unquestionable taeniolabidoid, possesses the gigantoprismatic prism with a slender but distinct seam. It indicates that either the seam has a mosaic distribution within taeniolabidoids or that some of non-seamed enamel reported in taeniolabidoid multituberculates could be an artifact that possibly resulted from inappropriate treatment of the specimens. As we experimented, the results of prism seams, particularly those that are as slender as those of *Lambdopsalis*, could be masked by artifacts derived from orientations of cross sections of the specimens or/and from differential etching owing to the enamel topography and acid strength. A more careful examination on the enamel seams in multituberculates is critical in discussing their evolution.

### Enamel spindles / tubules

Carlson and Krause [27] considered the enamel of *Lambdopsalis* to be among those of the multituberculates that have few or no enamel tubules, although there seem to be a couple of enamel tubules preserved in the enamel image of *Lambdopsalis* ([27], Fig 15B). Nonetheless, our study unequivocally demonstrates the presence of tubules in *Lambdopsalis*.

Sahni [88] considered that the multituberculate enamel near the EDJ contains abundant tubules that are restricted in interprismatic regions. The same author also observed that few tubules are present in the enamel near the tooth surface and, if present, they are restricted in the prism heads, not in the interprismatic region. This restricted condition of tubules is not found in our enamel study of *Lambdopsalis*. The presence or location of tubules in the enamel of *Lambdopsalis* does not seem to vary in any consistent manner, except that the number of enamel tubules decreases from the EDJ to the OES in all teeth we examined (Fig 7G). Therefore, in tangential sections at different depth of the enamel, the number of tubules will change considerably. The enamel tubules of *Lambdopsalis* are randomly present in the interprismatic regions and the prism heads, and are not restricted in the area near the EDJ or near the tooth surface. The one-to-one relationship between enamel tubule and prism head is absent in *Lambdopsalis*. Occasionally, more than one enamel tubules are present in a single prism core, as showed in Fig 7D.

Our investigation also shows that the continuous relationship across the EDJ and the similar shape and size, the dentine and enamel tubules are probably homologous features, formed by a similar mechanism during the development of the tooth. This also favors the odontoblastic origin of the enamel tubules, a subject that has been under rigorous debate in enamel developmental studies [62,89–93]. Whether the enamel tubules in primitive mammals, such as multituberculates, is homologous with the enamel spindles in extant mammals [87,89] remains open.

Carlson and Krause [27] considered that the irregular arrangement and the difficulty in imaging enamel tubules make it unsuitable as a character in phylogenetic reconstruction. However, the distinctive enamel tubules in *Lambdopsalis* let us support the view that the enamel tubule may become a useful enamel character in phylogenetic studies of mammals when additional samples from other multituberculates and mammals accumulate in the future [33].

### Deciduous enamel and implications

The diphyodont dentition with a sequential eruption pattern is one of the key features of mammals, which may have evolved in relation to lactation and parental care [94,95] as well as precise dental occlusion of mammals [96,97]. However, studies on deciduous teeth of multituberculates were few [98] and in our knowledge there is no literature on the enamel microstructures of multituberculate deciduous teeth so that we compare the microstructures of the deciduous and replacement incisors briefly here.

The two sets of teeth apparently reflect two ontogenetic stages of a mammal, and this is particularly so for *Lambdopsalis* because its large deciduous incisor (personal observation) may stay relatively long before it was replaced in the early stage of development. The specimens with deciduous incisors we examined probably represent the stage 1 and/or 2 in the six-stage model of tooth eruption sequence of multituberculates [98]. The permanent incisor differs from the deciduous one mainly in the following features: thicker enamel, prismatic covering more area of the tooth, and greater prism density but smaller prism size. In fact, the prism density of permanent incisors is greater than that of any permanent teeth in the three zones (Table 2). These differences show that the anti-abrasion performance of the permanent incisors should be much stronger than that of the deciduous tooth because the prismatic enamel was regarded as a characteristic to strengthen the wear resistance mechanically [99]. The enamel structure of the deciduous incisor suggests that *Lambdopsalis* had a soft diet period before the permanent incisors erupted.

Although multituberculates as to be viviparous have been inferred from the shape and size of the pelvic girdle [100], it is difficult, if possible at all, to know whether the juvenile multituberculates had an ontogenetic stage analogous to the lactation period in extent mammals. It is, however, highly probable that the food taken by the deciduous incisors was significantly different from that taken by the permanent incisors. This is also supported by the fact that the deciduous incisors are the only teeth that do not show pigmentation. Thus, the reddish pigmentation of the permanent teeth of *Lambdopsalis* is likely not owing to structural difference of the enamel; instead, it is more likely relevant to the chemical composition that may be resulted from change of dietary at certain stage of ontogeny.

For permanent incisors, the lower incisor has much thicker enamel than the upper one (Fig 16), which probably reflects the main functional role played by the lower incisor in food taking and burrowing [101].

## Conclusion

Because of the rich collection of *Lambdopsalis bulla*, a Late Paleocene taeniolabidoid multituberculate from Inner Mongolia, we are able to examine its enamel structures systematically. In permanent teeth, the general morphology of enamel microstructure shows a relative consistency in all teeth. The enamel has gigantoprisms that are radially oriented, of which most have the seam. While this observation favors a conclusion that enamel samples from any teeth in multituberculates may be used to help taxonomic identification and phylogenetic analysis, the enamel features should be used with caution because various variations do exist. In particular, the prism shape, size and density and enamel tubules vary in various degrees at different portions of a tooth and among different teeth of an individual animal. If preparation of specimens is not appropriate and the section planes cut improperly, the differences of these features can be exaggerated or even distorted.

In deciduous teeth, the enamel is significantly different from permanent teeth in being thinner, having aprismatic enamel that covers a larger portion of the tooth and completely lacking the pigmentation. The structural difference of the enamel suggests considerable dietary difference between juvenile and adult individuals of *Lambdopsalis*, which is probably true for other multituberculates.

The enamel structures of *Lambdopsalis* are systematically documented in this study, but whether the similar pattern throughout the entire dentition of the same species remains true in other multituberculates needs to be confirmed by similar surveys from other species of multituberculates. For the time being, however, we hope that the result of this study can provide a reference for studies that compare enamel microstructures of different teeth of various multituberculates.

## Supporting Information

S1 Table Measurements of the Prism size and prism density. SD, standard deviation about the prism diameter.
(XLS)
